# Fission yeast Duc1 links to ER–PM contact sites and influences PM lipid composition and cytokinetic ring anchoring

**DOI:** 10.1242/jcs.262347

**Published:** 2024-09-27

**Authors:** Alaina H. Willet, Joshua S. Park, Chloe E. Snider, Jingdian Jamie Huang, Jun-Song Chen, Kathleen L. Gould

**Affiliations:** Department of Cell and Developmental Biology, Vanderbilt University School of Medicine, Nashville, TN 37240, USA

**Keywords:** Fission yeast, Phosphatidylinositol (4,5)-bisphosphate, Phosphatidylinositol 4-phosphate 5-kinase, ER–PM contacts, Cytokinesis, Cytokinetic ring

## Abstract

Cytokinesis is the final stage of the cell cycle that results in the physical separation of daughter cells. To accomplish cytokinesis, many organisms build an actin- and myosin-based cytokinetic ring (CR) that is anchored to the plasma membrane (PM). Defects in CR–PM anchoring can arise when the PM lipid phosphatidylinositol (4,5)-bisphosphate [PI(4,5)P_2_] is depleted. In *Schizosaccharomyces pombe*, reduced PM PI(4,5)P_2_ results in a CR that cannot maintain a medial position and slides toward one cell end, resulting in two differently sized daughter cells. *S. pombe* PM PI(4,5)P_2_ is synthesized by the phosphatidylinositol 4-phosphate 5-kinase (PI5-kinase) Its3, but what regulates this enzyme to maintain appropriate PM PI(4,5)P_2_ levels in *S. pombe* is not known. To identify Its3 regulators, we used proximity-based biotinylation, and the uncharacterized protein Duc1 was specifically detected. We discovered that Duc1 decorates the PM except at the cell division site and that its unique localization pattern is dictated by binding to the endoplasmic reticulum (ER)–PM contact site proteins Scs2 and Scs22. Our evidence suggests that Duc1 also binds PI(4,5)P_2_ and helps enrich Its3 at the lateral PM, thereby promoting PM PI(4,5)P_2_ synthesis and robust CR–PM anchoring.

## Teaser

**Highlighted Article:** *Schizosaccharomyces pombe* Duc1 promotes correct localization of the phosphatidylinositol 4-phosphate 5-kinase Its3, normal plasma membrane PI(4,5)P_2_ levels and medial cell division.

## INTRODUCTION

To divide, many eukaryotes utilize an actin- and myosin-based cytokinetic ring (CR) comprising actin filaments, myosin motors and multiple other proteins (Cheffings et al., 2016; Gould, 2016; Mangione and Gould, 2019) that is attached to the plasma membrane (PM). One factor important for CR–PM linkage is the phosphoinositide (PIP) composition of the PM (Echard, 2012; Mangione and Gould, 2019). Out of the seven PIP species found in eukaryotic cells, PM phosphatidylinositol 4-phosphate (PI4P) and the most abundant PM PIP species, phosphatidylinositol 4,5-bisphosphate [PI(4,5)P_2_], are important for the fidelity of cell division in diverse organisms (Eggert et al., 2004; Emoto et al., 2005; Field et al., 2005; Janetopoulos et al., 2005; Snider et al., 2017, 2018; Wong et al., 2005).

In *Schizosaccharomyces pombe*, the CR normally remains anchored where it is assembled, in the middle of the longitudinal axis of the cell; thus, upon CR constriction and septation, two daughter cells of equal size are produced (Marks et al., 1986). In *S. pombe* cells lacking the scaffolding protein Efr3, the phosphatidylinositol 4-kinase (PI4-kinase) Stt4 is not localized to the PM and as a consequence, PM PI4P and PI(4,5)P_2_ levels are reduced (Snider et al., 2017). This results in the CR sliding away from center and subsequent off-center septation that can cut through the nucleus and chromosomes (Snider et al., 2017). Its3 is the PM-localized PI4P 5-kinase (PI5-kinase) responsible for converting PI4P to PI(4,5)P_2_ in *S. pombe* (Snider et al., 2017, 2018; Zhang et al., 2000). Like *efr3*Δ, *its3-1* cells display reduced PM PI(4,5)P_2_ levels, CR sliding and off-center septation (Snider et al., 2018). Mutants with diminished PI4P but normal PI(4,5)P_2_ levels (for example, *pik1-11* or *lsb6Δ*) do not have off-center septa, emphasizing the key role of PI(4,5)P_2_ in cytokinesis (Willet et al., 2023).

We previously identified a binding partner of *S. pombe* Its3, termed Opy1, that binds PI(4,5)P_2_ and serves as a useful PI(4,5)P_2_ sensor (Snider et al., 2020; Wei et al., 2023). However, PM PI4P and PI(4,5)P_2_ levels are not changed in *opy1Δ* cells, and we have found no evidence that Opy1 regulates Its3 activity *in vitro* or *in vivo* (Snider et al., 2020). Thus, if and how Its3 is regulated in *S. pombe* remains uncertain.

In this study, we identified a previously uncharacterized protein encoded by the open reading frame (ORF) SPCC594.01, which we named Duc1, based on its proximity to Its3 and Opy1 on the PM. We found that Duc1 localizes in PM puncta that we determined are sites where the endoplasmic reticulum (ER) connects to the PM. In yeast, non-vesicular lipid transport crucial for maintaining proper membrane lipid composition occurs at ER–PM contact sites (Zaman et al., 2020). The ER transmembrane VAMP-associated proteins (VAPs) Scs2 and Scs22 are integral to ER–PM connections (Zhang et al., 2012). Cells lacking VAPs have many fewer physical links between the ER and the PM, resulting in defects in cellular trafficking, lipid homeostasis and cell division site positioning (Zhang et al., 2012; Zhang and See, 2022). We determined that Duc1 binds directly to VAPs through its canonical diphenylalanines in an acidic tract (FFAT)-like motif. Furthermore, our data indicate that Duc1 binds PM PI(4,5)P_2_ at ER–PM contacts, where it promotes proper Its3 PM distribution, promotes PM PI(4,5)P_2_ synthesis and therefore contributes to proper CR–PM anchoring. Therefore, Duc1 contributes an additional mechanism by which ER–PM connections promote proper lipid homeostasis.

## RESULTS

### Identification of Duc1

To identify proteins associated with Its3 and/or its binding partner Opy1, the TurboID biotin ligase (BirA) was fused to the C terminus of each protein to facilitate biotinylation of proximal proteins *in vivo*. A streptavidin-based affinity purification procedure was used to isolate biotinylated proteins (Larochelle et al., 2019), and they were identified using mass spectrometry.

**Fig. 1.**
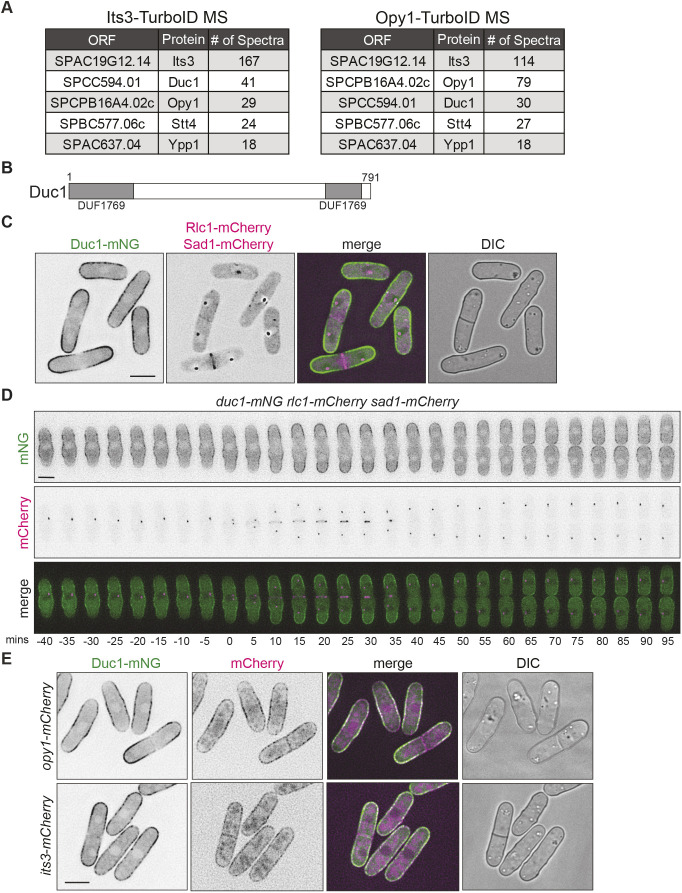
**Duc1 localizes to the cell cortex.** (A) Summary of data generated from proximity-dependent biotinylation experiments of Its3–TurboID and Opy1–TurboID. Number of spectra of selected proteins identified from mass spectrometry (MS) analysis is shown. (B) Schematic of Duc1, drawn to scale, with the split DUF1769 domain in gray. Numbers indicate amino acid residue positions. (C) Live-cell imaging of *duc1-mNG rlc1-mCherry sad1-mCherry*. (D) Live-cell time-lapse imaging of *duc1-mNG rlc1-mCherry sad1-mCherry* cells*.* Elapsed time is in minutes. Images were acquired every 5 min and time zero represents the first frame of SPB separation. (E) Live-cell imaging of Duc1–mNG with either Its3–mCherry or Opy1–mCherry. DIC, differential interference contrast image. Scale bars: 5 µm. Images in C–E are representative of two biological replicates.

Because many native *S. pombe* proteins are biotinylated (Larochelle et al., 2019; Tong, 2013), we performed additional proximity-dependent biotinylation experiments. The first control experiment consisted of expressing TurboID alone in cells and then purifying and identifying biotinylated proteins (Table S1). Second, we purified and identified biotinylated proteins from cells in which the essential type II myosin Myo2, which does not colocalize with either Its3 or Opy1, was tagged with TurboID (Table S1) (Kitayama et al., 1997; Snider et al., 2020). The Its3–TurboID experiment identified itself and Opy1, and the converse was true for the Opy1–TurboID experiment, validating the experimental approach (Fig. 1A; Table S1). Components of the PI4-kinase complex, Stt4 and Ypp1, were top hits in both the Opy1 and Its3 purifications (Fig. 1A; Table S1). Additionally, the non-essential uncharacterized protein encoded by SPCC594.01 emerged as a top hit in purifications from both *its3-TurboID* and *opy1-TurboID* cells (Fig. 1A; Table S1). SPCC594.01 was not identified in either of the control experiments (Table S1). We named this protein domain of unknown function at the cortex 1, Duc1, based on the characteristic localization pattern described below.

A BLASTP search (Altschul et al., 1990) identified proteins related to Duc1 in other fission yeast species, and we performed a sequence alignment of these Duc1-related proteins (Madeira et al., 2022) (Fig. S1). A schematic of the Duc1 protein highlights the bifurcated domain of unknown function (DUF)1769, which is conserved among these putative Duc1 orthologs (Fig. 1B; Fig. S1). The DUF1769 domain is present only in fungal proteins and has no ascribed function (Harris et al., 2022).

### Duc1 is a PM protein that is excluded from the cell division site

Because we identified Duc1 based on its proximity to Its3 and Opy1, both of which localize to the PM (Snider et al., 2017), we tested whether Duc1 also localized there. We did this in cells that also produced the CR marker Rlc1–mCherry (Le Goff et al., 2000) and the spindle pole body (SPB) marker Sad1–mCherry (Hagan and Yanagida, 1995) to monitor mitotic progression. Live-cell imaging of endogenously tagged Duc1–mNeonGreen (mNG) revealed that it localized along the cell cortex, although it was less abundant at cell tips during interphase and completely excluded from the medial cell region during cytokinesis (Fig. 1C). To define more precisely Duc1–mNG localization during the cell cycle, we used live-cell time-lapse imaging. This confirmed that Duc1–mNG localized along the central portion of the cell but not along cell tips during interphase (Fig. 1D). As cells progressed into mitosis, Duc1 re-localized towards cell tips and was excluded from the cell division site for the duration of mitosis and cytokinesis (Fig. 1D).

We next examined Duc1–mNG localization compared with Its3–mCherry and Opy1–mCherry. Although they all localized along the cortex (Fig. 1E) (Snider et al., 2018, 2020), Duc1 had a distinct localization pattern compared to that of Opy1 and Its3. This was especially apparent at the septum, where Opy1 and Its3 localized but Duc1 did not (Fig. 1E). Also, Opy1 and Its3 displayed localization along the cortex consistently throughout the cell cycle, whereas Duc1 accumulated at different cortical sites depending on the cell-cycle stage (Fig. 1E). These results indicate that Duc1 might be proximal to Its3 and Opy1 at the PM only at certain times.

### ER–PM contact sites influence Duc1 localization

**Fig. 2.**
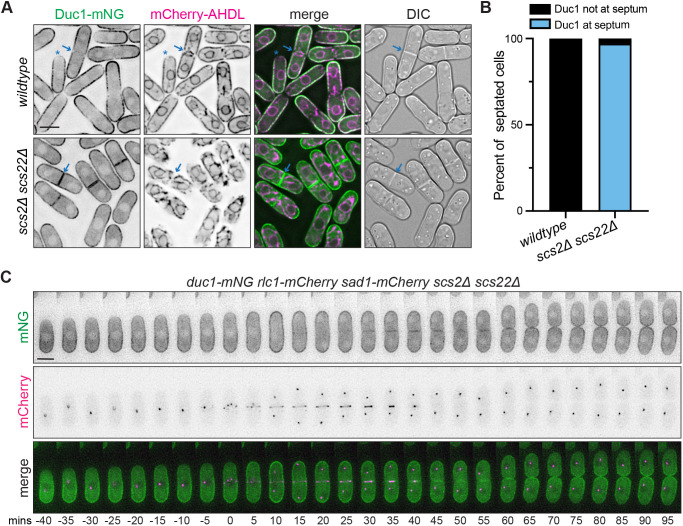
**Duc1 is a PM protein that is excluded from the cell division site.** (A) Live-cell imaging of Duc1–mNG and mCherry–AHDL in wild-type or *scs2Δ scs22Δ* cells. Blue arrows mark a septum, and blue asterisks mark a cell tip where localization is excluded. (B) Quantification of the frequency of Duc1 localization to the septum in septated cells from A. *n*=50 cells for each from two independent replicates. (C) Live-cell time-lapse imaging of *duc1-mNG rlc1-mCherry sad1-mcherry scs2Δ scs22Δ*. Elapsed time is in minutes. Images were acquired every 5 min and time zero represents the first frame of SPB separation. Images are representative of two biological replicates. DIC, differential interference contrast image. Scale bars: 5 µm.

Because the cortical ER underlies the PM in *S. pombe* (Pidoux and Armstrong, 1993) we wanted to verify that Duc1 is a PM protein rather than an ER protein. For this, we examined Duc1–mNG in cells that expressed the ER marker mCherry–AHDL and lacked Scs2 and Scs22, which form the major ER–PM linkages in *S. pombe*; in their absence ER–PM contact sites are disrupted and the ER partially separates from the PM (Zhang et al., 2012). In wild-type cells co-producing mCherry–AHDL, Duc1–mNG colocalized with the ER marker along the cortex and had overlapping zones of exclusion at cell tips and the cell division site (Fig. 2A). In *scs2Δ scs22Δ* cells, Duc1–mNG remained along the cell cortex, confirming its PM localization (Fig. 2A). However, we observed an intriguing change in Duc1 localization in *scs2Δ scs22Δ* cells. In contrast to its exclusion from the division site in wild-type cells, Duc1–mNG localized along the septa in *scs2Δ scs22Δ* cells (Fig. 2A,B). This observation was confirmed by live-cell time-lapse imaging with CR and SPB markers (Fig. 2C). In the absence of Scs2 and Scs22, Duc1–mNG localized to the septum after CR constriction commenced and remained there for the duration of cytokinesis (Fig. 2C).

**Fig. 3.**
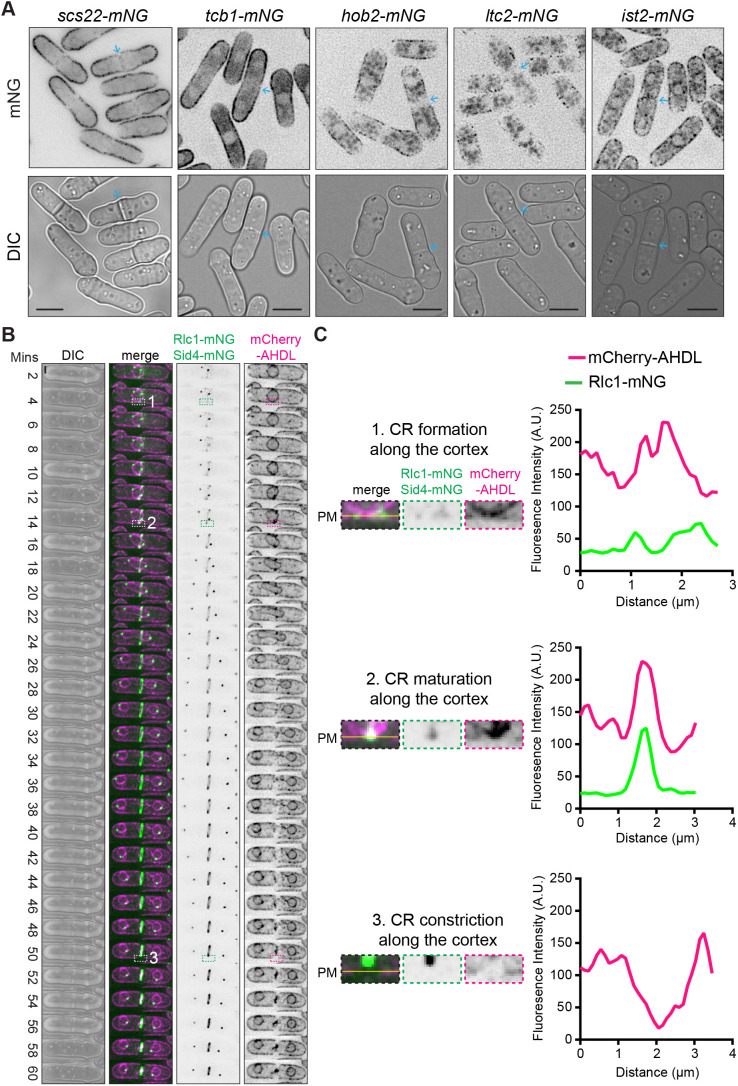
**ER–PM contact site proteins are excluded from the cell division site.** (A) Live-cell imaging of cells with the indicated mNG-tagged proteins. Blue arrows mark septa. Scale bars: 5 µm. Images are representative of two biological replicates. (B) Live-cell time-lapse imaging of cells expressing mCherry–AHDL, Rlc1–mNG and Sid4–mNG. Elapsed time is shown relative to SPB separation. Numbered boxes indicate regions shown in C. Scale bar: 2 µm. (C) Left, zoomed in views of the frames of the montage indicated in boxes numbered 1–3 from B. The yellow lines correspond to the position of the PM. Right, fluorescence line scan analysis of the frames on the right from the montages in B. Fluorescence intensity was plotted versus distance. Images and line scans in B and C are representative of two biological replicates. A.U., arbitrary units; DIC, differential interference contrast image.

Like Duc1, Scs2 is excluded from the cell division site as CR formation occurs (Zhang and See, 2022). In addition to the VAPs Scs2 and Scs22, other *S. pombe* proteins form ER–PM contact sites, including the LAM-family protein Ltc2, the Anoctamin (TMEM16) homolog Ist2, the Hobbit ortholog Hob2, and the tricalbins Tcb1, Tcb2 and Tcb3 (Zaman et al., 2020). These proteins contain at least one ER transmembrane helix and additional modules that directly or indirectly link these proteins to the PM (Zaman et al., 2020). Despite the existence of several protein families important for linking the ER to the PM, VAPs are the prominent linkages in *S. pombe*. To see whether these other ER–PM contact site proteins had a similar localization pattern to Duc1 and Scs2, we imaged Scs22, Tcb1, Hob2, Ltc2 or Ist2 tagged with mNG. Live-cell imaging revealed that they all localized either uniformly or in a punctate pattern along the lateral cell cortex, and all but Ist2 lacked any detectable localization at the cell division site (Fig. 3A). Furthermore, live-cell time-lapse imaging of *mCherry-AHDL rlc1-mNG* cells revealed that the ER underlying the PM rearranged during cell division. Line scan analysis showed that the cortical ER was relatively uniform underneath the PM during CR formation and then became enriched at overlapping medial cortical sites surrounding the nucleus during CR maturation (Fig. 3B,C). During CR constriction, the ER was absent from the PM at the cell division site (Fig. 3B,C), indicating that ER–PM contact sites are largely excluded from the PM adjacent to and underlying the septum during cell division.

To dissect the contribution of each class of ER–PM contact to Duc1–mNG localization, we analyzed Duc1–mNG distribution in *hob2Δ*, *ltc2Δ*, *ist2Δ*, and *tcb1Δ tcb2Δ tcb3Δ* strains. We found that Duc1–mNG was excluded from the cell division site in all four strains, as in wild-type cells (Fig. S2A). We also examined whether Scs2 or Scs22 alone was responsible for preventing Duc1 division site localization but, as in wild-type cells, Duc1–mNG was excluded from the cell division site in both single deletion strains (Fig. S2B). Thus, Duc1 localization is specifically controlled by the combination of VAPs Scs2 and Scs22.

In *scs2Δ scs22Δ* cells, a majority of ER–PM contacts are disrupted (Zhang et al., 2012) Thus, to distinguish whether Duc1 exclusion from the cell division site depends on the Scs2 and Scs22 proteins or more generally on ER–PM contact sites, we utilized an artificial ER–PM tether where an ER transmembrane anchor from Tts1 is fused to mCherry and the PM-binding domain of Osh3 (Zhang et al., 2012). We expressed this tether (TM–mCherry–PH_Osh3_) or just the Tts1 transmembrane anchor fused to mCherry (TM–mCherry) in *scs2Δ scs22Δ duc1-mNG* cells. Artificially tethering the ER to the PM in *scs2Δ scs22Δ* cells with TM–mCherry–PH_Osh3_ did not restore Duc1–mNG exclusion from the septa (Fig. S2C). Thus, we conclude that Duc1 exclusion from the cell division site depends specifically on Scs2 and Scs22 and not on ER–PM contact sites per se.

### Duc1 directly associates with Scs2 and Scs22

**Fig. 4.**
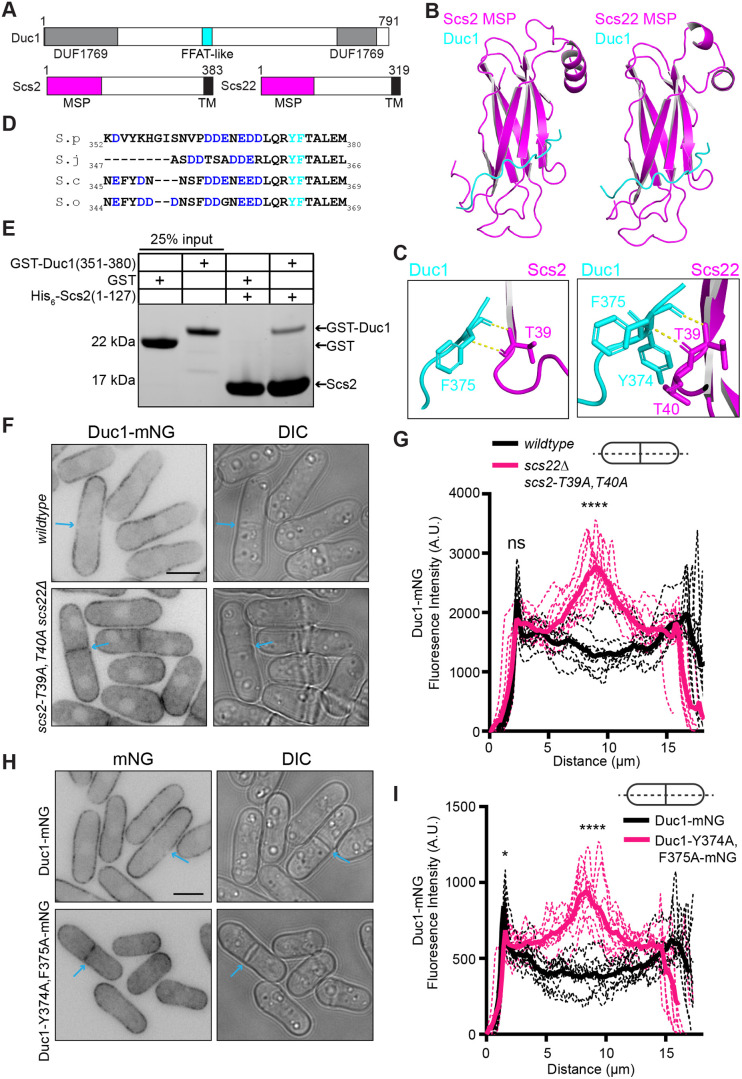
**Duc1 is predicted to directly bind Scs2 and Scs22.** (A) Schematic, drawn to scale, of Duc1, Scs2 and Scs22. The DUF1769 domain is gray, the FFAT-like sequence is cyan, the MSP domains are magenta, and the transmembrane (TM) domains are black. Numbers indicate amino acid residue positions. (B) AlphaFold2 prediction of the Duc1–MSP complexes. Full-length Duc1 and either Scs2 (amino acids 1–132; left) or Scs22 (amino acids 1–125; right) were modeled. Duc1 residues 364–376 are cyan and MSP domains are magenta. (C) Zoomed-in views of the Duc1–Scs2 (left) and Duc1–Scs22 (right) interaction interfaces. Residue predicted to directly bind are labeled. (D) Sequence alignment of the indicated Duc1 proteins. Acidic residues are in blue, and Y374 and F375 are cyan. Numbers indicate amino acid residue positions. *S. pombe* (S.p), *Schizosaccharomyces japonicus* (S.j), *Schizosaccharomyces octosporus* (S.o) and *Schizosaccharomyces cryophilus* (S.c) sequences are shown. (E) *In vitro* binding assay with bead-bound His_6–_Scs2(1–127) incubated with GST or GST–Duc1(351–380). Samples were washed three times and resolved by SDS-PAGE. The gel was stained with Coomassie Blue to visualize proteins. Gel shown is representative of three experiments. (F) Live-cell imaging of Duc1–mNG in wild-type or *scs2-T39A, T40A scs22Δ* cells. Blue arrows indicate a septum. (G) Fluorescence line scans drawn across the long axis of cells as in F. *n*=10 cells for each from two independent replicates. Solid lines represent the mean and dotted lines are the individual line traces. Wild-type cells versus scs22Δ *scs2-T39A, T40A* cells at first peak, 2.3 μm distance, *P*=0.051. Wild-type cells versus *scs22Δ scs2-T39A, T40A* cells at a medial distance, 8.9 μm, *P*<0.0001. ****P<0.0001; ns, not significant (unpaired, two-tailed Student's *t*-test). (H) Live-cell imaging of cells expressing Duc1–mNG or Duc1-Y374A, F375A–mNG. Blue arrows indicate a septum. (I) Fluorescence line scans drawn across the long axis of cells as in H. *n*=10 cells for each from two independent replicates. Solid lines represent the mean and dotted lines are the individual line traces. Duc1–mNG versus Duc1–Y374A, F375A at first peak, 1.58 μm distance, *P*<0.05. Duc1–mNG versus Duc1–Y374A, F375A at a medial distance, 8.35 μm, *P*<0.0001. *P<0.05; ****P<0.0001 (unpaired, two-tailed Student's *t*-test). Scale bars: 5 µm. A.U., arbitrary units; DIC, differential interference contrast image.

Given the specific control of Duc1 localization by Scs2 and Scs22, we examined the potential of direct interactions. Typically, the major sperm protein (MSP) domains of VAPs bind an FFAT motif or an FFAT-like motif within protein partners (Loewen et al., 2003; Loewen and Levine, 2005; Slee and Levine, 2019). Using Alphafold2 (Jumper et al., 2021; Mirdita et al., 2022; Varadi et al., 2022), we found that Duc1–Scs2 and Duc1–Scs22 interactions were predicted. Duc1 was also identified by mass spectrometry in large-scale purifications of full-length and MSP domain-only versions of Scs2 and Scs22 (Hoh et al., 2024). An unstructured region outside of the Duc1 DUF1769 domain was predicted by AlphaFold2 to interact with the MSP domains of Scs2 and Scs22 (Fig. 4A,B; Fig. S3A) via conserved Duc1 aromatic residues Y374 and F375, which are downstream of an acidic tract (Fig. 4C,D). This Duc1 sequence is reminiscent of a FFAT motif as it also contains the typical alanine and glutamate at the second and fourth positions downstream of the aromatic residues, respectively (Loewen et al., 2003). AlphaFold2 also predicted that T39 and/or T40 within Scs2 and Scs22, which are typical FFAT-binding residues, contact Duc1 (Fig. 4C) (Furuita et al., 2010; Kaiser et al., 2005; Loewen and Levine, 2005). To determine whether Scs2 and Duc1 directly interact, we recombinantly produced a His-tagged version of the Scs2 MSP domain [His_6_–Scs2(1–127)] and a GST-tagged version of the FFAT-like sequence of Duc1 [GST–Duc1(351–380)]. *In vitro* binding assays confirmed that the Scs2 MSP domain directly bound Duc1 but not GST alone (Fig. 4E).

To test the hypothesis that Duc1 directly interacts with Scs2 and Scs22 to restrict its cell division site localization, we constructed an *scs2* allele that encodes T39A and T40A mutations at the endogenous locus (*scs2-T39A, T40A*). These mutations are expected to disrupt FFAT interactions (Loewen and Levine, 2005; Manford et al., 2012). Imaging of the mCherry–AHDL ER marker in *scs2-T39A, T40A scs22Δ* cells revealed that most ER–PM contact sites were maintained (Fig. S3B), which is consistent with a previous report (Hoh et al., 2024). Analysis of Duc1–mNG localization showed that, unlike in wild-type cells, Duc1–mNG localized at the septum in *scs2-T39A, T40A scs22Δ* cells (Fig. 4F). Line scan analysis revealed that the lateral cortical intensity of Duc1–mNG in *scs2-T39A, T40A scs22Δ* cells was unchanged, but the septum intensity was enriched by ∼2.2-fold compared to that in wild-type cells (Fig. 4F,G). Reciprocally, we also constructed a *duc1* allele at the endogenous locus containing mutations within the FFAT-like motif (Y374A and F375A) and tagged it with mNG (Duc1*-*Y374A, F375A–mNG). As would be anticipated if these mutations successfully disrupted Duc1 association with Scs2 and Scs22, Duc1*-*Y374A, F375A–mNG localized at the division site (Fig. 4H). Line scan analysis confirmed a ∼2.5-fold enrichment of Duc1*-*Y374A, F375A–mNG at the septum and a ∼15% reduction along the cortex compared to wild-type Duc1–mNG (Fig. 4H,I). These data support the proposal that Duc1–VAP interactions dictate Duc1 PM distribution.

### *In vivo* data suggest that Duc1 is a PI(4,5)P_2_-binding protein

**Fig. 5.**
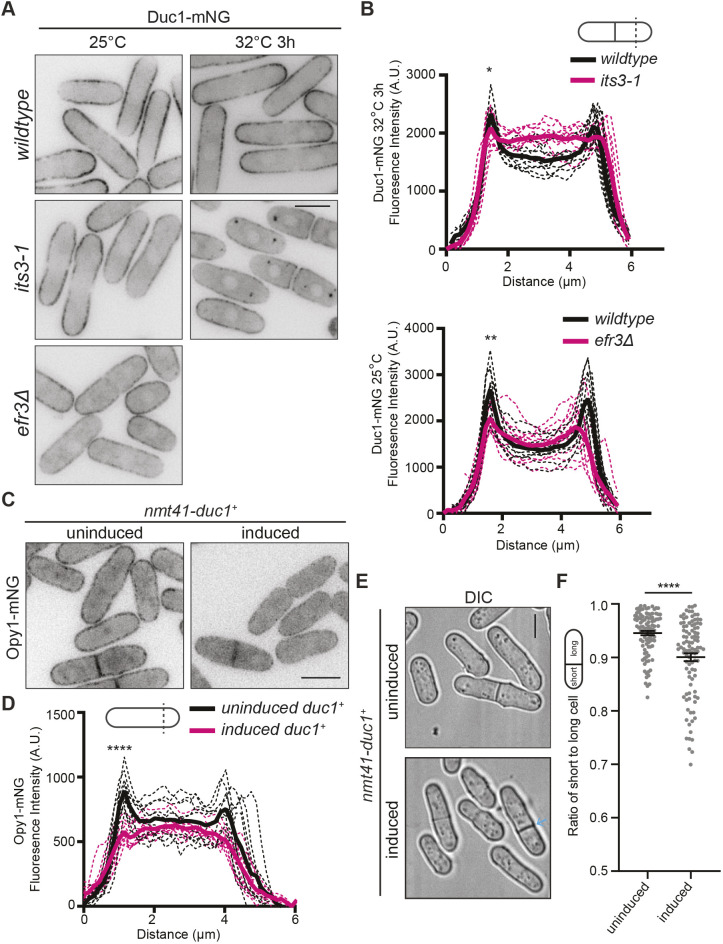
**Duc1 behaves like a PI(4,5)P_2_-binding protein.** (A) Live-cell imaging of Duc1–mNG in the indicated genetic backgrounds grown at the indicated temperatures. (B) Fluorescence line scans drawn across the short axis of cells as in A. *n*=10 cells for each from two independent replicates. Solid lines represent the mean and dotted lines are the individual line traces. Wild-type cells versus *its3-1* cells at first peak, 1.44 μm distance, *P<*0.05. Wild-type cells versus *efr3Δ* cells at first peak, 1.58 μm distance, *P<*0.01. *P<0.05; **P<0.01 (unpaired, two-tailed Student's *t*-test). (C) Live-cell imaging of Opy1–mNG with *nmt41-duc1^+^* expression either repressed (uninduced; grown with thiamine) or induced (grown without thiamine for 24 h) prior to imaging. (D) Fluorescence line scans drawn across the short axis of cells as in C. *n*=10 cells for each from two independent replicates. Solid lines represent the mean and dotted lines are the individual line traces. Uninduced versus induced at first peak, 1.15 μm distance, *P<*0.0001. ****P<0.0001 (unpaired, two-tailed Student's *t*-test). (E) Live-cell imaging of the indicated strains. *duc1^+^* expression was either repressed (uninduced; grown with thiamine) or induced (grown without thiamine for 30 h) prior to imaging. The blue arrow marks an off-center septum. (F) Quantification of the ratio of short to long daughter cell length for strains as in E. Horizontal line marks the mean, and error bars represent the s.e.m. *n*≥99 for each from two independent replicates. ****P<0.0001 (unpaired, two-tailed Student's *t*-test). Scale bars: 5 µm. A.U., arbitrary units; DIC, differential interference contrast image.

We next examined the nature of Duc1 PM association. Because Duc1 is in proximity to Its3, which synthesizes PI(4,5)P_2_, we tested whether Duc1 requires PI(4,5)P_2_ for PM localization. To test this, we analyzed Duc1–mNG localization in mutants with reduced levels of PM PI4P and PI(4,5)P_2_. We found that Duc1–mNG PM localization along the lateral cell cortex was diminished by ∼25% in *efr3Δ* cells compared to that in wild-type cells (Fig. 5A,B)*.* Duc1–mNG levels were also reduced at the lateral PM in *its3-1* cells at a semi-restrictive temperature compared to levels in wild-type cells (Fig. 5A,B). These results are consistent with Duc1 requiring PM PI(4,5)P_2_ for PM localization.

Opy1 PM localization depends on PI(4,5)P_2_, and Opy1 itself binds directly to liposomes containing PI(4,5)P_2_ *in vitro* (Snider et al., 2020). Thus, we reasoned that if Duc1 associates with PM PI(4,5)P_2_, then overproduction of Duc1 would displace Opy1–mNG from the PM and vice versa. Live-cell imaging revealed that overproduction of Duc1 indeed reduced levels of Opy1–mNG at the PM by ∼35% (Fig. 5C,D). The converse was also true because overproduction of Opy1 resulted in a 20% reduction in cortical Duc1–mNG compared to that in control cells (Fig. S3C,D). Furthermore, we noticed that overproduction of Duc1, like overproduction of Opy1 (Snider et al., 2020), resulted in cells with off-center septation (Fig. 5E,F), which is consistent with both of these proteins associating with PI(4,5)P_2_ and outcompeting CR-anchoring proteins for a limiting amount of PI(4,5)P_2_.

### Duc1 influences PM lipid composition and the position of septation

**Fig. 6.**
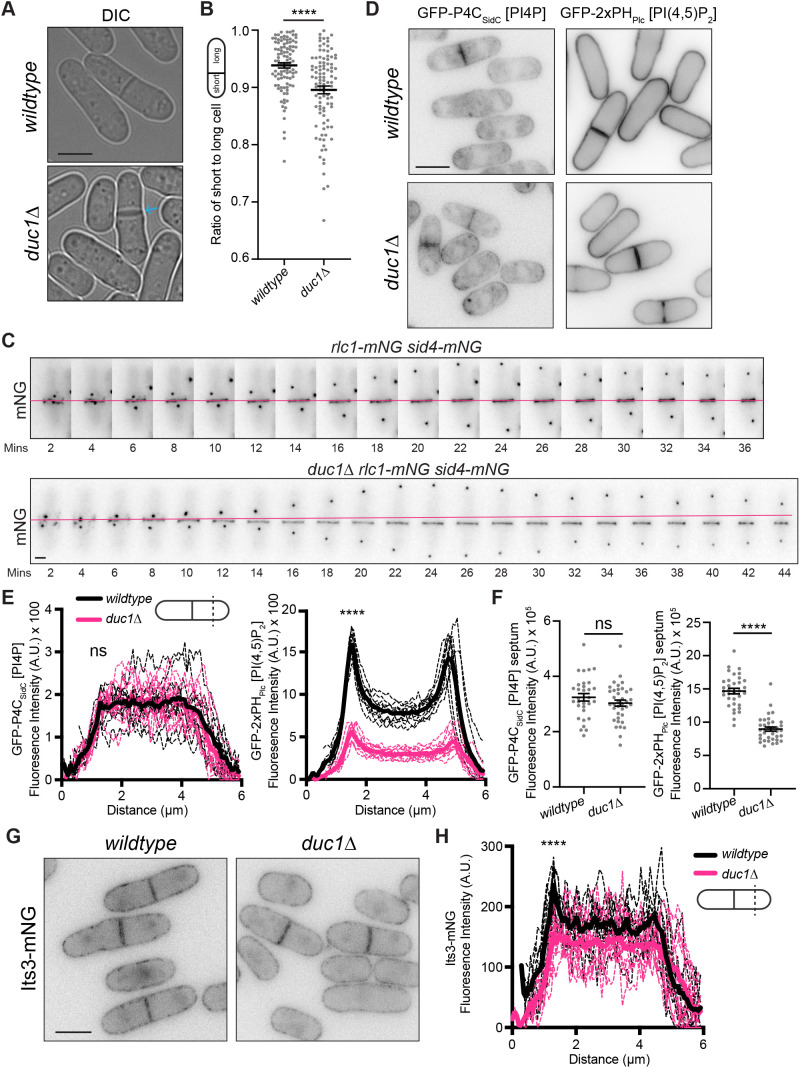
**CR sliding is observed in *duc1*Δ mutant cells.** (A) Live-cell imaging of the indicated strains. The blue arrow marks a septum. (B) Quantification of the ratio of short to long daughter cell length for strains as in A. Horizontal line marks the mean, and error bars represent the s.e.m. *n*=100 for each from two independent replicates. ****P<0.0001 (unpaired, two-tailed Student's *t*-test). (C) Live-cell time-lapse imaging of Rlc1–mNG and Sid4–mNG in wild-type or *duc1*Δ cells. Images were acquired every two minutes. Numbers indicate minutes elapsed; magenta line indicates the position where the CR was formed. Data is representative of four independent replicates. (D) Live-cell imaging of wild-type and *duc1Δ* cells expressing GFP–P4C_SidC_ or GFP–2×PH_Plc_. (E) Fluorescence line scans drawn across the short axis of cells as in D. *n*=10 cells for each from three independent replicates. Solid lines represent the mean and dotted lines are the individual line traces. GFP–P4C_SidC_ wild-type cells versus *duc1Δ* cells at first peak, 1.29 μm distance, *P*=0.754. GFP–2×PH_Plc_ wild-type cells versus *duc1Δ* cells at first peak, 1.51 μm distance, *P*<0.0001. ****P<0.0001; ns, not significant (unpaired, two-tailed Student's *t*-test). (F) Fluorescence intensity of GFP–P4C_SidC_ or GFP–2×PH_Plc_ at the septum of wild-type or *duc1Δ* cells from D. Data is from three independent replicates and *n*≥30 for each. Horizontal line marks the mean, and error bars represent the s.e.m. Wild-type versus *duc1Δ* GFP–P4C_SidC_, *P*=0.23. Wild-type versus *duc1Δ* GFP–2×PH_Plc_, *P*<0.0001. ****P<0.0001; ns, not significant (unpaired, two-tailed Student's *t*-test). (G) Live-cell imaging of Its3–mNG in wild-type or *duc1Δ* cells. (H) Fluorescence line scans drawn across the short axis of cells as in G. *n*=10 cells for each from two independent replicates. Wild-type versus *duc1Δ* cells at first peak, 1.29 μm distance, *P*<0.0001. ****P<0.0001 (unpaired, two-tailed Student's *t*-test). Scale bars: 5 µm in A,D,G; 2 µm in C. A.U., arbitrary units; DIC, differential interference contrast image.

*duc1* is not essential for viability, and *duc1Δ* cells grow similarly to wild-type cells at a variety of temperatures (Fig. S4A) (Hayles et al., 2013; Kim et al., 2010). The localizations of Scs2–mNG, Scs22–mNG and mCherry–AHDL all appeared unchanged in *duc1Δ* cells compared to their localization in wild-type cells (Fig. S4B). However, a quantification of the ratio of short-to-long *duc1Δ* cells at septation demonstrated that a significant fraction of *duc1Δ* cells divided asymmetrically (Fig. 6A,B). Off-center septation can result from off-center CR assembly or, alternatively, medial assembly and subsequent sliding to an off-center position. To determine which was the case in *duc1Δ* cells, we performed live-cell time-lapse imaging of cells expressing Rlc1–mNG and Sid4–mNG. Wild-type cells formed a medial CR that slid in one of 13 cells examined, but *duc1Δ* cells formed a medial CR that slid along the cortex towards one cell end in 12 of 15 cells observed (Fig. 6C). Thus, Duc1 promotes proper CR anchoring.

CR anchoring defects can arise from altered PM PIP composition (Snider et al., 2017; 2018). We therefore used lipid biosensors to compare the levels of PM PIPs in wild-type cells to those in *duc1Δ* cells. There was no significant change in lateral cortex or septum PI4P levels, as determined by the GFP–P4C_SidC_ biosensor (Fig. 6D–F). In contrast, lateral PM and septum PI(4,5)P_2_ levels, indicated by the GFP–2×PH_Plc_ biosensor, were 36% and 48%, respectively, in *duc1Δ* cells compared to levels in wild-type cells (Fig. 6D–F). Thus, the CR sliding observed in *duc1*Δ cells is likely due to a reduction of lateral PM PI(4,5)P_2_ levels.

We reasoned that reduced levels of PI(4,5)P_2_ could be caused by a change in the localization or activity of the enzymes involved in its synthesis. We therefore analyzed the localization of Efr3–mNG and Its3–mNG in wild-type and *duc1Δ* cells. We detected no change in lateral cortex Efr3–mNG but a ∼30% decrease in lateral cortex Its3–mNG in *duc1Δ* cells compared to levels in wild-type cells (Fig. 6G,H; Fig. S4C,D). There was no change in Its3–mNG septum intensity in *duc1Δ* cells compared to that in wild-type cells, as expected (Fig. S4E).

We next examined whether *duc1Δ* cells had genetic interactions with other mutants with similar defects. We found that *duc1Δ* had no genetic interaction with *opy1Δ* or *efr3Δ* but interacted negatively with *its3-1* (Fig. S4A), consistent with Duc1 promoting Its3 PM localization.

**Fig. 7.**
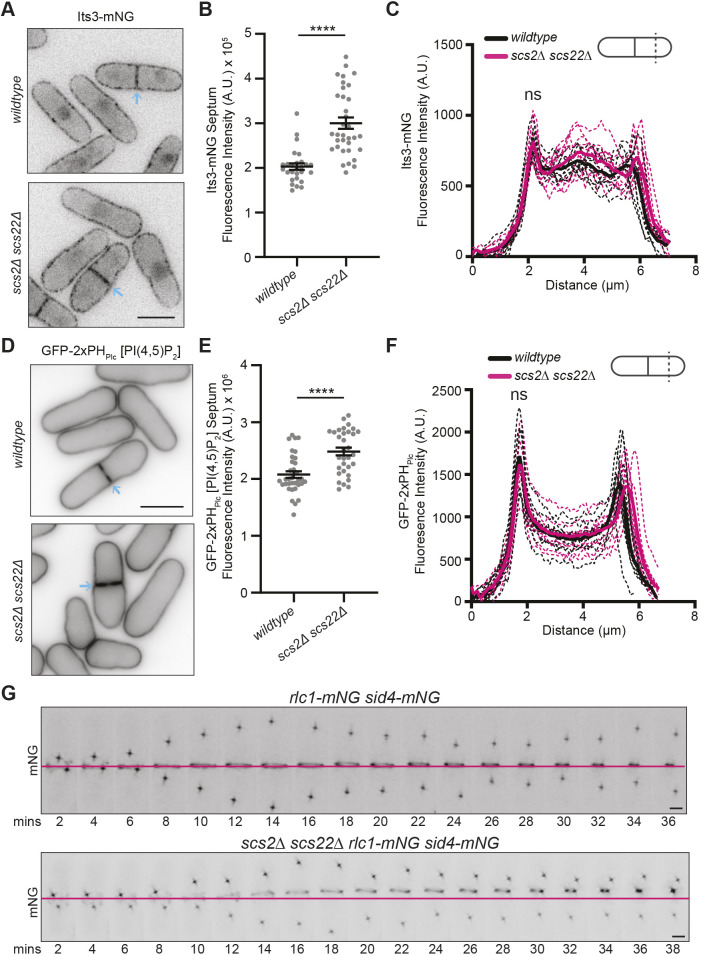
**Cells with disrupted ER–PM contact sites have CR anchoring defects.** (A) Live-cell imaging of Its3–mNG in wild-type and *scs2Δ scs22Δ* cells. Blue arrows indicate septa. (B) Quantification of the fluorescence intensity of Its3–mNG at the septum in strains from A. *n*≥27 cells for each from two independent replicates. Horizontal line marks the mean, and error bars represent the s.e.m. ****P<0.0001 (unpaired, two-tailed Student's *t*-test). (C) Fluorescence line scans drawn across the short axis of cells as in A. *n*=10 cells for each from two independent replicates. Solid lines represent the mean and dotted lines are the individual line traces. Wild-type versus *scs2Δ scs22Δ* Its3–mNG at first peak, 2.16 μm distance, *P*=0.84. ns, not significant (unpaired, two-tailed Student's *t*-test). (D) Live-cell imaging of wild-type and *scs2Δ scs22Δ* cells expressing GFP–2×PH_Plc_. Blue arrows indicate septa. (E) Quantification of the fluorescence intensity of GFP–2×PH_Plc_ at the septum in strains from D. *n*≥32 cells for each from three independent replicates. Horizontal line marks the mean, and error bars represent the s.e.m. ****P<0.0001 (unpaired, two-tailed Student's *t*-test). (F) Fluorescence line scans drawn across the long axis of cells as in D. *n*=10 cells for each from three independent replicates. Solid lines represent the mean and dotted lines are the individual line traces. Wild-type versus *scs2Δ scs22Δ* GFP–2×PH_Plc_ at first peak, 1.72 μm distance, *P*=0.49. ns, not significant (unpaired, two-tailed Student's *t*-test). (G) Live-cell time-lapse imaging of Rlc1–mNG and Sid4–mNG in wild-type or *scs2Δ scs22Δ* cells. Images were acquired every two minutes. Numbers indicate minutes elapsed; magenta line indicates the position where the CR was formed. Data is representative of three independent experiments. Scale bars: 5 µm in A and D, 2 µm in G. A.U., arbitrary units.

Lastly, we wanted to better understand the function of tethering Duc1 at ER–PM contact sites. Because Duc1 promotes Its3 function at the PM, we reasoned that mis-localized Duc1 might alter Its3 localization and thus also the distribution of PM PI(4,5)P_2_. To test this, we analyzed Its3–mNG localization in wild-type cells and *scs2Δ scs22Δ* cells, and we found that the level of Its3–mNG was increased at the septum in *scs2Δ scs22Δ* cells by ∼50% compared to that in wild-type cells, without a change in the lateral cortex levels (Fig. 7A–C). It has previously been reported that levels of PI4P are increased and levels of PI(4,5)P_2_ are unchanged at the cell cortex in *scs2Δ scs22Δ* cells compared to those in wild-type cells (Zhang et al., 2012). We confirmed a lack of change in lateral cortical PI(4,5)P_2_ signal but found that the PI(4,5)P_2_ signal was increased at the septum by ∼20% in *scs2Δ scs22Δ* cells compared to that in wild-type cells (Fig. 7D–F). These data support the idea that Duc1–VAP interactions help maintain the proper distribution of PM PI(4,5)P_2_.

It has previously been reported that *scs2Δ scs22Δ* cells have off-center septa (Zhang et al., 2012). Although some of these cells form the CR at an off-center position (Zhang et al., 2012), given the changes in PM PI(4,5)P_2_ due to changes in Duc1 localization, we asked whether the CRs were properly anchored in these cells. Live-cell time-lapse imaging of Rlc1–mNG Sid4–mNG in wild-type cells and *scs2Δ scs22Δ* cells revealed that whereas two of 50 wild-type cells displayed CR sliding, CRs slid toward one cell end in eight of 28 *scs2Δ scs22Δ* cells (Fig. 7G). Thus, both CR formation and anchoring are disrupted in cells lacking ER–PM contact sites.

## DISCUSSION

In this study, we identified the previously uncharacterized *S. pombe* protein Duc1 based on its proximity to the PM-localized PI-5 kinase Its3 and the Its3 binding partner Opy1. We determined that Duc1 promotes the proper PM localization of Its3 and therefore functions to maintain proper lateral PM lipid composition, CR anchoring and medial septation.

We also found that Duc1 binds the ER–PM contact site proteins Scs2 and Scs22 via a canonical mechanism wherein MSP domains in the VAPs bind an FFAT-like motif in their partners (Loewen et al., 2003; Loewen and Levine, 2005; Slee and Levine, 2019). We further determined that these interactions prevent Duc1 from localizing at the cell division site. Interestingly, Scs2 itself is excluded from the division site as the CR forms (Zhang and See, 2022), and we found that all but one of the other known ER–PM contact site proteins are also excluded from the cell division site. In accord, we found that the cortical ER is remodeled during cell division so that it concentrates underneath the forming CR and is then excluded from the cell division site during cytokinesis, and this change likely aides in efficient septation. Fascinatingly, in plants, the presence of ER membrane during cytokinesis inhibits complete abscission and allows the formation of specialized structures called plasmodesmata, which interconnect the cytoplasm of neighboring cells (Li et al., 2023 preprint). We speculate that in scenarios where complete abscission is required for proper cell function, there might be an active mechanism to spatially restrict the ER network from the cell division site. It will be exciting to explore what controls the exclusion of ER–PM contact site proteins and cortical ER from the *S. pombe* division site.

Although the PM distribution of Duc1 is governed by VAP interactions, Duc1 still localizes along the entire PM in cells lacking Scs2 and Scs22. This suggests that Duc1 has intrinsic membrane-binding properties. In support of Duc1 directly binding PM PI(4,5)P_2_, overproduction of Duc1 displaces the PI(4,5)P_2_-binding protein Opy1 (Snider et al., 2020), and conversely, overproduction of Opy1 displaced Duc1 from the PM. Furthermore, overproduction of Duc1 resulted in cells with off-center septa, a phenotype also observed in cells overproducing Opy1, in which CRs slide away from the cell center (Snider et al., 2020). We have previously proposed that this CR phenotype is achieved when overproduced PI(4,5)P_2_-binding proteins displace an ensemble of proteins that rely on PI(4,5)P_2_ to anchor the CR (Snider et al., 2020). Our findings, together with the result that robust Duc1 PM localization depends on proper PM PI(4,5)P_2_ levels, lead us to propose that Duc1 likely associates with PI(4,5)P_2_ directly. It is still uncertain whether the Duc1 DUF1769 domain and/or the unstructured portion of Duc1 that bisects the two halves of the domain support direct PI(4,5)P_2_ association. Both Duc1 regions contain patches of basic residues, which are typical PI(4,5)P_2_-binding residues, and thus further structure–function analysis would be required to gain additional insight into the mechanism of Duc1 PM binding.

Interestingly, we saw an enrichment of Its3 and the lipid PI(4,5)P_2_ at the septum in cells with diminished ER–PM contacts. PI(4,5)P_2_ is not the only lipid that is impacted when ER–PM contact sites are disrupted. In *Saccharomyces cerevisiae* cells with disrupted ER–PM contacts, the phosphatidylinositol 4-phosphatase is no longer juxtaposed to the PM and, as a result, PM PI4P levels are increased (Stefan et al., 2011). Similarly, in *S. pombe* cells lacking Scs2 and Scs22, PM PI4P levels are elevated (Zhang et al., 2010). Furthermore, many VAP-interacting proteins, such as the oxysterol-binding homology proteins, are lipid transfer proteins that exchange lipids between membranes (Zaman et al., 2020). PI4P, PI(4,5)P_2_ and phosphatidylserine are substrates of this family of proteins and are exchanged between membranes via non-vesicular transport (Li et al., 2021; Maeda et al., 2013; Moser von Filseck et al., 2015), highlighting the potential for a wide range of lipid composition defects when ER–PM contacts are compromised. Our data indicate that Duc1 represents another example by which ER–PM contact sites promote proper lipid composition within the cell, and it will be interesting to learn the mechanism by which Duc1 controls Its3.

## MATERIALS AND METHODS

### Yeast methods

*S. pombe* strains (Table S2) were grown in yeast extract (YE) medium or Edinburgh minimal medium (EMM) plus appropriate supplements (Forsburg and Rhind, 2006; Moreno et al., 1991). *duc1*, *scs2*, *scs22*, *tcb1*, *hob2*, *ltc2* and *ist2* were tagged at the 3′ end of their ORFs with the sequences encoding *mCherry:kanMX6*, *mNG:kanMX6* or *mNG:hphMX6* using pFA6 cassettes as previously described (Bähler et al., 1998; Wach et al., 1994). *its3*, *opy1* and *myo2* were endogenously tagged at the 3′ end of their ORFs with the sequences encoding *TurboID-V5:kanMX6* (Larochelle et al., 2019). Integration of tags was verified using whole-cell PCR and/or microscopy. *S. pombe* lithium acetate transformation methods followed previously described protocols (Keeney and Boeke, 1994). Introduction of tagged loci into genetic backgrounds was accomplished using standard *S. pombe* mating, sporulation and tetrad dissection techniques (Forsburg and Rhind, 2006). Fusion proteins were expressed from their native promoters at their normal chromosomal loci.

The gene deletion of *tcb1Δ::kanMX6* was made as previously described (Chen et al., 2015). The gene deletions of *tcb2Δ::ura4^+^* and *hob2Δ::ura4^+^* were made by cloning 300 bp of the respective 5′ untranslated regions (UTRs) into the BamHI/PstI sites and 300 bp of the respective 3′ UTRs into the KpnI/XhoI sites within a pIRT2 plasmid (Hindley et al., 1987) containing the *ura4^+^* gene within the PstI/KpnI sites. A PCR product was amplified that contained the UTRs and *ura4^+^*; this DNA was transformed into wild-type cells, and colonies were selected on EMM agar plates lacking uracil. Colonies were verified by whole-cell PCR.

The *scs2-T39A, T40A* allele was constructed by synthesizing a version of the *scs2* gene that encodes the T39A and T40A mutations with 300 bp of both the 5′ and 3′ flanks (Integrated DNA technologies). This fragment was inserted into the pIRT2 PstI site using Gibson cloning. Correct cloning was confirmed by sequencing. The plasmid was transformed into *scs2Δ::ura4^+^* cells, and colonies were selected with 5-fluoroorotic acid (5-FOA, 1.5 mg/ml), confirmed with whole-cell PCR and sequencing from the genome.

Duc1 genomic DNA was also cloned into the NdeI/BamHI sites of pREP41 (Basi et al., 1993) using Gibson assembly and sequenced to confirm. The *duc1-F374A, Y375A* strain was made by cloning the *duc1* ORF and 300 bp of the 3′ and 5′ UTRs into the Pst1 site of pIRT2 using Gibson assembly. The relevant Duc1 mutations were added by site-directed mutagenesis and confirmed with sequencing. The pIRT2-Duc1-F374A,Y375A plasmid was transformed into *duc1::ura4^+^* cells, and integrants were selected on YE plates with 5-FOA. The *duc1-F374A, Y375A* allele was confirmed with sequencing. Protein expression from plasmids was induced by switching cells from EMM containing 5 µM thiamine to EMM lacking thiamine for 24–30 h. The *scs2Δ scs22Δ* strain was a gift from Dr Snezhana Oliferenko, The Francis Crick Institute, London, UK.

### Microscopy methods

Yeast for live-cell imaging were grown at 25°C in YE medium. Live-cell images of *S. pombe* cells were acquired using a either (1) a personal DeltaVision microscope system (Leica Microsystems), which includes an Olympus IX71 microscope, 60× NA 1.42 Plan Apochromat and 100× NA 1.40 U-Plan S-Apochromat objectives, live-cell and standard filter wheel sets, a pco.edge 4.2 sCMOS camera and softWoRx imaging software; or (2) a Zeiss Axio Observer inverted epifluorescence microscope with a Zeiss 63× oil (1.46 NA) objective, Zeiss ZEN 3.0 (Blue edition) software and an Axiocam 503 monochrome camera (Zeiss). All images acquired with the DeltaVision microscope and used for representative images in Figs 1, 2 and 3B were deconvolved with 10 iterations. Representative images in Fig. 3B are maximum projections of the full *z*-stack. Images in all other figures except Figs 5E and 6A are maximum projections of the three medial *z* slices. Figs 5E and 6A are a single medial *z*-slice. *Z*-stacks were acquired with 0.5 µm spacing for a total of 5 µm distance.

For all line scans, a sum projection of three medial *z*-slices of non-deconvolved images were used. Background was subtracted and intensity measurements were plotted against the distance. The line scans were aligned by the first peak in fluorescence intensity.

Time-lapse imaging was performed using an CellASIC ONIX microfluidics perfusion system (Millipore Sigma) with the DeltaVision microscope. Cells were loaded into Y04C plates for 5 s at 8 psi (∼55 kPa), and YE liquid medium flowed into the chamber at 5 psi (∼34.5 kPa) throughout imaging. For time-lapse images, *z*-series optical sections were taken at 0.5 µm spacing, and images were acquired every 5 min.

### Protein methods

Pellets containing 1.5×10^10^ cells/ml from *its3-TurboID-V5:kanMX6*, *opy1-TurboID-V5:kanMX6*, *myo2-TurboID-V5:kanMX6* and wild-type strains were collected and frozen in liquid nitrogen. Cells were lysed by bead disruption in urea lysis buffer (8 M urea, 50 mM Tris-HCl pH 8.5, 1 mM PMSF, 1.3 mM benzamidine). Lysates were cleared by centrifugation at 2060 ***g*** for 5 min. Streptavidin Plus UltraLink Resin (Thermo Fisher Scientific) was added to the lysates before nutating the mixture for 2 h at 25°C. A single culture was used of each strain and the experiment was performed once.

### Mass spectrometric analysis

Mass spectrometric analysis was performed as described previously (Beckley et al., 2015) with modifications. Streptavidin beads from TurboID purifications were washed with Tris–urea buffer (100 mM Tris-HCl, pH 8.5, 2 M urea) three times. Proteins were reduced with 3 mM Tris(2-carboxyethyl)phosphine hydrochloride, alkylated with 10 mM chloroacetamide, and digested with trypsin (1 μg of Trypsin Gold, Promega) at 37°C overnight with shaking. The digest supernatant and washes were combined, concentrated and desalted with a Pierce C18 spin column (Thermo Fisher Scientific). Peptides were resuspended in 0.1% formic acid and loaded onto a 26 cm column [consisting of 3 cm of 5 µm Jupiter C-18 (Phenomenex), 3 cm of 5 µm SCX (Phenomenex) and 20 cm of 3 µm Jupiter C-18 in 100 µm fused silica capillary tubing] with a pressure cell and then separated and analyzed by three-phase multidimensional protein identification technology (MudPIT) on a Velos linear trap quadrupole (LTQ) mass spectrometer (Thermo Fisher Scientific) coupled to a nanoHPLC (NanoAcquity; Waters Corporation). The NanoAcquity autosampler was used to inject 2 μl of varying concentrations of ammonium acetate (0, 10, 25, 50, 100, 200, 300, 400, 600, 800, 1000 and 5000 mM) for 11 salt elution steps. Each injection was followed by elution of peptides with a 2–40% acetonitrile gradient (60 min) except the first and last injections, in which a 2–90% acetonitrile gradient was used. One full precursor mass MS scan (400–2000 mass-to-charge ratio) and five tandem MS (MS2) scans of the most abundant ions detected in the precursor MS scan under dynamic exclusion were performed. Ions with a neutral loss of 98 Da (singly charged), 49 Da (doubly charged), or 32.7 Da (triply charged) from the parent ions during MS2 were subjected to MS3 fragmentation.

RAW files containing more than 20 peaks were converted to DTA files using Scansifter software (Ma et al., 2011) (v2.1.25). Each DTA file was searched using the SEQUEST algorithm (Thermo Fisher Scientific; version 27, rev. 12). SEQUEST was set up to search the pombe_contams_20151012_20151012_rev database (downloaded from PomBase in October 2015 with common contaminants added and all sequences reversed, 10390 entries in total). Variable modifications (C+57, M+16, [STY]+80, [STY]−18), strict tryptic cleavage, <10 missed cleavages, fragment mass tolerance: 0.00 Da (this results in 0.5 Da tolerance in SEQUEST), and parent mass tolerance: 2.5 Da were allowed. Peptide identifications were assembled and filtered in Scaffold (v4.7.5, Proteome Software) using the following criteria: minimum of 99% protein identification probability; minimum of two unique peptides; minimum of 95% peptide identification probability. These filtering criteria were used to achieve false discovery rates less than 1%.

### Recombinant protein production

The *scs2* sequence encoding amino acid residues 1–127 was amplified from *S. pombe* genomic DNA and cloned into the NdeI and BamHI sites of pET15b (Novagen) by Gibson assembly. Similarly, the *duc1* sequence encoding amino acid residues 351–380 was amplified from genomic DNA and cloned into pGEX-6P-1 (Cytiva). His_6_–Scs2(1–127), GST and GST–Duc1(351–380) were produced in Rosetta2(DE3)pLysS bacteria (Novagen) grown in Terrific broth (23.6 g/l yeast extract, 11.8 g/l tryptone, 9.4 g/l K_2_HPO_4_, 2.2 g/l KH_2_PO_4_, 4 ml/l glycerol) supplemented with 100 μg/ml ampicillin and 34 μg/ml chloramphenicol by incubating on ice for 15 min, adding 0.4 mM isopropyl β-D-1-thiogalactopyranoside (IPTG) (Fisher Scientific; BP1755), and incubating the cells for 16–18 h at 18°C. Lysates were sonicated three times for 30 s, with a 30 s pause between sonications (Sonic Dismembrator Model F60, Fisher Scientific; power 15 W). Lysates were cleared for 15 min at 10,000 ***g***. Cleared lysate was then used in a batch purification protocol.

GST fragments were purified with GST-bind resin (Millipore Sigma, 70541) in 4.3 mM sodium phosphate pH 7.3, 137 mM NaCl, 2.7 mM KCl, 1 mM DTT, 0.1% NP40, 1 mM PMSF, 1.3 mM benzamidine and protease inhibitor cocktail (Roche). Resin was washed three times with buffer and eluted with glutathione (50 mM Tris-HCl pH 8.0, 10 mM glutathione).

His_6_–Scs2(1–127) was purified by incubating with cOmplete His-Tag resin (Roche, 5893682001) in 20 mM Tris-HCl pH 7.4 and 150 mM NaCl, 0.1% NP-40, 1 mM DTT, 1 mM PMSF, 2 mM benzamidine and protease inhibitor cocktail (Roche) for 1 h at 4°C. Resin was then washed three times with buffer.

### *In vitro* binding assay

10 µg recombinant GST or GST–Duc1(351–380) was incubated in 500 µl His wash buffer (20 mM Tris-HCl pH 7.4 and 150 mM NaCl, 0.1% NP-40) with 2–3 µg of bead-bound His_6_–Scs2(1–127) for 30 min at 4°C. The beads were washed three times with 1 ml His wash buffer. Bead-bound proteins were resuspended in 20 µl 2× SDS sample buffer, heated at 95°C for 2 min, and separated on a 4–12% NuPAGE Bis-Tris gel with MOPS buffer (NP0322BOX; Invitrogen, Carlsbad, CA, USA). Proteins were visualized by Coomassie blue staining.

### Protein structure prediction and sequence alignment

Protein structure predictions were generated with the ColabFold interface to the AlphaFold2 pipeline on the Colab platform (AlphaFold2.ipynb; Jumper et al., 2021; Mirdita et al., 2022; Varadi et al., 2022). Protein sequence alignments were performed using Clustal Omega (Madeira et al., 2022; Sievers and Higgins, 2021).

### Statistical analysis

All statistical analyses were performed in Prism 8 (GraphPad Software). No data were excluded from the analysis.

## Supplementary Material

10.1242/joces.262347_sup1 Supplementary information

Table S1. Proteins identified by LC-MS/MS in Its3-TurboID, Opy1-TurboID, and Myo2-TurboID. Shown in the table are the ORF numbers (ID), protein names, and protein descriptions. Also indicated are the Total Spectral Counts (TSC) of proteins from both the bait protein-TurboID and the control-TurboID, Only proteins with a minimum of two spectral counts are shown and proteins identified in control-TurboID are listed at the end of the table in grey-shaded text.

## References

[JCS262347C1] Altschul, S. F., Gish, W., Miller, W., Myers, E. W. and Lipman, D. J. (1990). Basic local alignment search tool. *J. Mol. Biol.* 215, 403-410. 10.1016/S0022-2836(05)80360-2 2231712

[JCS262347C2] Bähler, J., Wu, J. Q., Longtine, M. S., Shah, N. G., McKenzie, A., Steever, A. B., Wach, A., Philippsen, P. and Pringle, J. R. (1998). Heterologous modules for efficient and versatile PCR-based gene targeting in Schizosaccharomyces pombe. *Yeast (Chichester, England)* 14, 943-951. 10.1002/(SICI)1097-0061(199807)14:10<943::AID-YEA292>3.0.CO;2-Y 9717240

[JCS262347C54] Basi, G., Schmid, E. and Maundrell, K. (1993). TATA box mutations in the Schizosaccharomyces pombe nmt1 promoter affect transcription efficiency but not the transcription start point or thiamine repressibility. *Gene* 123, 131-136. 10.1016/0378-1119(93)90552-e 8422997

[JCS262347C3] Beckley, J. R., Chen, J.-S., Yang, Y., Peng, J. and Gould, K. L. (2015). A degenerate cohort of yeast membrane trafficking DUBs mediates cell polarity and survival. *Mol. Cell. Proteomics* 14, 3132-3141. 10.1074/mcp.M115.050039 26412298 PMC4762628

[JCS262347C4] Cheffings, T. H., Burroughs, N. J. and Balasubramanian, M. K. (2016). Actomyosin ring formation and tension generation in eukaryotic cytokinesis. *Curr. Biol.* 26, R719-R737. 10.1016/j.cub.2016.06.071 27505246

[JCS262347C5] Chen, J.-S., Beckley, J. R., McDonald, N. A., Ren, L., Mangione, M., Jang, S. J., Elmore, Z. C., Rachfall, N., Feoktistova, A., Jones, C. M. et al. (2015). Identification of new players in cell division, DNA damage response, and morphogenesis through construction of Schizosaccharomyces pombe deletion strains. *G3 (Bethesda)* 5, 361-370. 10.1534/g3.114.015701 PMC4349090 25552606

[JCS262347C6] Echard, A. (2012). Phosphoinositides and cytokinesis: the “PIP” of the iceberg. *Cytoskeleton (Hoboken, N.J.)* 69, 893-912. 10.1002/cm.21067 23012232

[JCS262347C7] Eggert, U. S., Kiger, A. A., Richter, C., Perlman, Z. E., Perrimon, N., Mitchison, T. J. and Field, C. M. (2004). Parallel chemical genetic and genome-wide RNAi screens identify cytokinesis inhibitors and targets. *PLoS Biol.* 2, e379. 10.1371/journal.pbio.0020379 15547975 PMC528723

[JCS262347C8] Emoto, K., Inadome, H., Kanaho, Y., Narumiya, S. and Umeda, M. (2005). Local change in phospholipid composition at the cleavage furrow is essential for completion of cytokinesis. *J. Biol. Chem.* 280, 37901-37907. 10.1074/jbc.M504282200 16162509

[JCS262347C9] Field, C. M., Coughlin, M., Doberstein, S., Marty, T. and Sullivan, W. (2005). Characterization of anillin mutants reveals essential roles in septin localization and plasma membrane integrity. *Development* 132, 2849-2860. 10.1242/dev.01843 15930114

[JCS262347C10] Forsburg, S. L. and Rhind, N. (2006). Basic methods for fission yeast. *Yeast (Chichester, England)* 23, 173-183. 10.1002/yea.1347 16498704 PMC5074380

[JCS262347C11] Furuita, K., Jee, J., Fukada, H., Mishima, M. and Kojima, C. (2010). Electrostatic interaction between oxysterol-binding protein and VAMP-associated protein A revealed by NMR and mutagenesis studies. *J. Biol. Chem.* 285, 12961-12970. 10.1074/jbc.M109.082602 20178991 PMC2857075

[JCS262347C12] Gould, G. W. (2016). Animal cell cytokinesis: the role of dynamic changes in the plasma membrane proteome and lipidome. *Semin. Cell Dev. Biol.* 53, 64-73. 10.1016/j.semcdb.2015.12.012 26721337

[JCS262347C13] Hagan, I. and Yanagida, M. (1995). The product of the spindle formation gene sad1+ associates with the fission yeast spindle pole body and is essential for viability. *J. Cell Biol.* 129, 1033-1047. 10.1083/jcb.129.4.1033 7744953 PMC2120497

[JCS262347C14] Harris, M. A., Rutherford, K. M., Hayles, J., Lock, A., Bähler, J., Oliver, S. G., Mata, J. and Wood, V. (2022). Fission stories: using PomBase to understand Schizosaccharomyces pombe biology. *Genetics* 220, iyab222. 10.1093/genetics/iyab222 35100366 PMC9209812

[JCS262347C15] Hayles, J., Wood, V., Jeffery, L., Hoe, K.-L., Kim, D.-U., Park, H.-O., Salas-Pino, S., Heichinger, C. and Nurse, P. (2013). A genome-wide resource of cell cycle and cell shape genes of fission yeast. *Open Biol.* 3, 130053. 10.1098/rsob.130053 23697806 PMC3866870

[JCS262347C55] Hindley, J., Phear, G., Stein, M. and Beach, D. (1987). *suc1^+^* encodes a predicted 13-kilodalton protein that is essential for cell viability and is directly Involved in the division cycle of Schizosaccharomyces pombe. *Mol. Cell Biol.* 7, 504-511. 10.1128/mcb.7.1.504-511.1987 3031478 PMC365094

[JCS262347C16] Hoh, K. L., Mu, B., See, T., Ng, A. Y. E., Ng, A. Q. E. and Zhang, D. (2024). VAP-mediated membrane-tethering mechanisms implicate ER-PM contact function in pH homeostasis. *Cell Reports* 43, 114592. 10.1016/j.celrep.2024.114592 39110593

[JCS262347C17] Janetopoulos, C., Borleis, J., Vazquez, F., Iijima, M. and Devreotes, P. (2005). Temporal and spatial regulation of phosphoinositide signaling mediates cytokinesis. *Dev. Cell* 8, 467-477. 10.1016/j.devcel.2005.02.010 15809030

[JCS262347C18] Jumper, J., Evans, R., Pritzel, A., Green, T., Figurnov, M., Ronneberger, O., Tunyasuvunakool, K., Bates, R., Žídek, A., Potapenko, A. et al. (2021). Highly accurate protein structure prediction with AlphaFold. *Nature* 596, 583-589. 10.1038/s41586-021-03819-2 34265844 PMC8371605

[JCS262347C19] Kaiser, S. E., Brickner, J. H., Reilein, A. R., Fenn, T. D., Walter, P. and Brunger, A. T. (2005). Structural basis of FFAT motif-mediated ER targeting. *Structure (London, England: 1993)* 13, 1035-1045. 10.1016/j.str.2005.04.010 16004875

[JCS262347C20] Keeney, J. B. and Boeke, J. D. (1994). Efficient targeted integration at leu1-32 and ura4-294 in Schizosaccharomyces pombe. *Genetics* 136, 849-856. 10.1093/genetics/136.3.849 8005439 PMC1205890

[JCS262347C21] Kim, D.-U., Hayles, J., Kim, D., Wood, V., Park, H.-O., Won, M., Yoo, H.-S., Duhig, T., Nam, M., Palmer, G. et al. (2010). Analysis of a genome-wide set of gene deletions in the fission yeast Schizosaccharomyces pombe. *Nat. Biotechnol.* 28, 617-623. 10.1038/nbt.1628 20473289 PMC3962850

[JCS262347C22] Kitayama, C., Sugimoto, A. and Yamamoto, M. (1997). Type II myosin heavy chain encoded by the myo2 gene composes the contractile ring during cytokinesis in Schizosaccharomyces pombe. *J. Cell Biol.* 137, 1309-1319. 10.1083/jcb.137.6.1309 9182664 PMC2132538

[JCS262347C23] Larochelle, M., Bergeron, D., Arcand, B. and Bachand, F. (2019). Proximity-dependent biotinylation mediated by TurboID to identify protein-protein interaction networks in yeast. *J. Cell Sci.* 132, jcs232249. 10.1242/jcs.232249 31064814

[JCS262347C24] Le Goff, X., Motegi, F., Salimova, E., Mabuchi, I. and Simanis, V. (2000). The S. pombe rlc1 gene encodes a putative myosin regulatory light chain that binds the type II myosins myo3p and myo2p. *J. Cell Sci.* 113, 4157-4163. 10.1242/jcs.113.23.4157 11069761

[JCS262347C25] Li, C., Qian, T., He, R., Wan, C., Liu, Y. and Yu, H. (2021). Endoplasmic reticulum–plasma membrane contact sites: regulators, mechanisms, and physiological functions. *Front. Cell Dev. Biol.* 9, 627700. 10.3389/fcell.2021.627700 33614657 PMC7889955

[JCS262347C26] Li, Z. P., Moreau, H., Petit, J. D., Souza-Moraes, T., Smokvarska, M., Perez-Sancho, J., Petrel, M., Decoeur, F., Brocard, L., Chambaud, C. et al. (2023). Plant plasmodesmata bridges form through ER-driven incomplete cytokinesis. *BioRxiv* 2023.12.12.571296. 10.1101/2023.12.12.571296 39480927

[JCS262347C27] Loewen, C. J. R. and Levine, T. P. (2005). A highly conserved binding site in vesicle-associated membrane protein-associated protein (VAP) for the FFAT motif of lipid-binding proteins. *J. Biol. Chem.* 280, 14097-14104. 10.1074/jbc.M500147200 15668246

[JCS262347C28] Loewen, C. J. R., Roy, A. and Levine, T. P. (2003). A conserved ER targeting motif in three families of lipid binding proteins and in Opi1p binds VAP. *EMBO J.* 22, 2025-2035. 10.1093/emboj/cdg201 12727870 PMC156073

[JCS262347C29] Ma, Z.-Q., Tabb, D. L., Burden, J., Chambers, M. C., Cox, M. B., Cantrell, M. J., Ham, A.-J. L., Litton, M. D., Oreto, M. R., Schultz, W. C. et al. (2011). Supporting tool suite for production proteomics. *Bioinformatics* 27, 3214-3215. 10.1093/bioinformatics/btr544 21965817 PMC3208394

[JCS262347C30] Madeira, F., Pearce, M., Tivey, A. R. N., Basutkar, P., Lee, J., Edbali, O., Madhusoodanan, N., Kolesnikov, A. and Lopez, R. (2022). Search and sequence analysis tools services from EMBL-EBI in 2022. *Nucleic Acids Res.* 50, W276-W279. 10.1093/nar/gkac240 35412617 PMC9252731

[JCS262347C31] Maeda, K., Anand, K., Chiapparino, A., Kumar, A., Poletto, M., Kaksonen, M. and Gavin, A.-C. (2013). Interactome map uncovers phosphatidylserine transport by oxysterol-binding proteins. *Nature* 501, 257-261. 10.1038/nature12430 23934110

[JCS262347C32] Manford, A. G., Stefan, C. J., Yuan, H. L., Macgurn, J. A. and Emr, S. D. (2012). ER-to-plasma membrane tethering proteins regulate cell signaling and ER morphology. *Dev. Cell* 23, 1129-1140. 10.1016/j.devcel.2012.11.004 23237950

[JCS262347C33] Mangione, M. C. and Gould, K. L. (2019). Molecular form and function of the cytokinetic ring. *J. Cell Sci.* 132, jcs226928. 10.1242/jcs.226928 31209062 PMC6602304

[JCS262347C34] Marks, J., Hagan, I. M. and Hyams, J. S. (1986). Growth polarity and cytokinesis in fission yeast: the role of the cytoskeleton. *J. Cell Sci. Suppl.* 5, 229-241. 10.1242/jcs.1986.supplement_5.15 3477553

[JCS262347C35] Mirdita, M., Schütze, K., Moriwaki, Y., Heo, L., Ovchinnikov, S. and Steinegger, M. (2022). ColabFold: making protein folding accessible to all. *Nat. Methods* 19, 679-682. 10.1038/s41592-022-01488-1 35637307 PMC9184281

[JCS262347C36] Moreno, S., Klar, A. and Nurse, P. (1991). Molecular genetic analysis of fission yeast Schizosaccharomyces pombe. *Methods Enzymol.* 194, 795-823. 10.1016/0076-6879(91)94059-l 2005825

[JCS262347C37] Moser von Filseck, J., Čopič, A., Delfosse, V., Vanni, S., Jackson, C. L., Bourguet, W. and Drin, G. (2015). Phosphatidylserine transport by ORP/Osh proteins is driven by phosphatidylinositol 4-phosphate. *Science*, 349, 432-436. 10.1126/science.aab1346 26206936

[JCS262347C38] Pidoux, A. L. and Armstrong, J. (1993). The BiP protein and the endoplasmic reticulum of Schizosaccharomyces pombe: fate of the nuclear envelope during cell division. *J. Cell Sci.* 105, 1115-1120. 10.1242/jcs.105.4.1115 8227200

[JCS262347C39] Sievers, F. and Higgins, D. G. (2021). The clustal omega multiple alignment package. *Method. Mol. Biol. (Clifton, N.J.)* 2231, 3-16. 10.1007/978-1-0716-1036-7_1 33289883

[JCS262347C40] Slee, J. A. and Levine, T. P. (2019). Systematic prediction of FFAT motifs across eukaryote proteomes identifies nucleolar and eisosome proteins with the predicted capacity to form bridges to the endoplasmic reticulum. *Contact (Thousand Oaks (Ventura County, Calif.))* 2, 1-21. 10.1177/2515256419883136 31777772 PMC6881177

[JCS262347C41] Snider, C. E., Willet, A. H., Chen, J. S., Arpag, G., Zanic, M. and Gould, K. L. (2017). Phosphoinositide-mediated ring anchoring resists perpendicular forces to promote medial cytokinesis. *J. Cell Biol.* 216, 3041-3050. 10.1083/jcb.201705070 28784611 PMC5626552

[JCS262347C42] Snider, C. E., Willet, A. H., Brown, H. T. and Gould, K. L. (2018). Analysis of the contribution of phosphoinositides to medial septation in fission yeast highlights the importance of PI(4,5)P2 for medial contractile ring anchoring. *Mol. Biol. Cell* 29, 2148-2155. 10.1091/mbc.E18-03-0179 29975157 PMC6249800

[JCS262347C43] Snider, C. E., Willet, A. H., Brown, H. T., Chen, J.-S., Evers, J. M. and Gould, K. L. (2020). Fission yeast Opy1 is an endogenous PI(4,5)P2 sensor that binds to the phosphatidylinositol 4-phosphate 5-kinase Its3. *J. Cell Sci.* 133, jcs247973. 10.1242/jcs.247973 33172987 PMC7725598

[JCS262347C56] Stefan, C. J., Manford, A. G., Baird, D., Yamada-Hanff, J., Mao, Y. and Emr, S. D. (2011). Osh proteins regulate phosphoinositide metabolism at ER-plasma membrane contact sites. *Cell*, 144, 389-401. 10.1016/j.cell.2010.12.034 21295699

[JCS262347C44] Tong, L. (2013). Structure and function of biotin-dependent carboxylases. *Cell. Mol. Life Sci.* 70, 863-891. 10.1007/s00018-012-1096-0 22869039 PMC3508090

[JCS262347C45] Varadi, M., Anyango, S., Deshpande, M., Nair, S., Natassia, C., Yordanova, G., Yuan, D., Stroe, O., Wood, G., Laydon, A. et al. (2022). AlphaFold Protein structure database: massively expanding the structural coverage of protein-sequence space with high-accuracy models. *Nucleic Acids Res.* 50, D439-D444. 10.1093/nar/gkab1061 34791371 PMC8728224

[JCS262347C46] Wach, A., Brachat, A., Pöhlmann, R. and Philippsen, P. (1994). New heterologous modules for classical or PCR-based gene disruptions in Saccharomyces cerevisiae. *Yeast (Chichester, England)* 10, 1793-1808. 10.1002/yea.320101310 7747518

[JCS262347C47] Wei, W., Zheng, B., Zheng, S., Wu, D., Chu, Y., Zhang, S., Wang, D., Ma, X., Liu, X., Yao, X. et al. (2023). The Cdc42 GAP Rga6 promotes monopolar outgrowth of spores. *J. Cell Biol.* 222, e202202064. 10.1083/jcb.202202064 36355349 PMC9652770

[JCS262347C48] Willet, A. H., Turner, L. A., Park, J. S., Ren, L., Snider, C. E. and Gould, K. L. (2023). Characterization of Pik1 function in fission yeast reveals its conserved role in lipid synthesis and not cytokinesis. *J. Cell Sci.* 136, jcs261415. 10.1242/jcs.261415 37815455 PMC10629694

[JCS262347C49] Wong, R., Hadjiyanni, I., Wei, H.-C., Polevoy, G., McBride, R., Sem, K.-P. and Brill, J. A. (2005). PIP2 hydrolysis and calcium release are required for cytokinesis in Drosophila spermatocytes. *Curr. Biol.* 15, 1401-1406. 10.1016/j.cub.2005.06.060 16085493

[JCS262347C50] Zaman, M. F., Nenadic, A., Radojičić, A., Rosado, A. and Beh, C. T. (2020). Sticking with It: ER-PM membrane contact sites as a coordinating nexus for regulating lipids and proteins at the cell cortex. *Front. Cell Dev. Biol.* 8, 675. 10.3389/fcell.2020.00675 32793605 PMC7387695

[JCS262347C51] Zhang, D. and See, T. (2022). Coordinated cortical ER remodeling facilitates actomyosin ring assembly. *Curr. Biol.* 32, 2694-2703.e4. 10.1016/j.cub.2022.04.086 35609605

[JCS262347C58] Zhang, D., Vjestica, A. and Oliferenko, S. (2010). The cortical ER network limits the permissive zone for actomyosin ring assembly. *Curr. Biol.* 20, 1029-1034. 10.1016/j.cub.2010.04.017 20434336

[JCS262347C52] Zhang, Y., Sugiura, R., Lu, Y., Asami, M., Maeda, T., Itoh, T., Takenawa, T., Shuntoh, H. and Kuno, T. (2000). Phosphatidylinositol 4-phosphate 5-kinase its3 and calcineurin Ppb1 coordinately regulate cytokinesis in fission yeast. *J. Biol. Chem.* 275, 35600-35606. 10.1074/jbc.M005575200 10950958

[JCS262347C53] Zhang, D., Vjestica, A. and Oliferenko, S. (2012). Plasma membrane tethering of the cortical ER necessitates its finely reticulated architecture. *Curr. Biol.* 22, 2048-2052. 10.1016/j.cub.2012.08.047 23041194

