# Supplementary material for: Fission yeast Duc1 links to ER–PM contact sites and influences PM lipid composition and cytokinetic ring anchoring
Source: J Cell Sci. 2024 Sep 27;137(18):jcs262347. doi: 10.1242/jcs.262347 (PMC11449445; doi:10.1242/jcs.262347)
Supplement: Supplementary information [file joces-137-262347-s1.pdf]

```
S.j  MKVRVRVGPDPEHTQIAWVNHEGRPTEIDGPYWVGRILVRIRDFSGITPDGSAPKRDSEY 60
S.p  MKLKVSIGCDAQHTQIAWVNYEGRPTEVDGPLWVGRILVRVRDFDGFTPDGSPPKRDSEY 60
S.c  MKLKVNIGSDPGHTQIAWVNHEGRPTEVDGPFWVGRILVRVRDFDGITPDGSPPKSDSIY 60
S.o  MKLKVNVGPDPGHTQIAWVNHEGRPTEVDGPYWVGRILVRVRDFDGVTPDGSPPKADSIY 60
     **::* :* *  ********:******:*** ********:***.*.***** ** ** *

S.j  FKGRSRKFQIQAEGRFKKEYNGDQVIYGTQFDHMISHFPESAFRAGMRIAKYIDPAVYYD 120
S.p  FRGRSRKFQIQAEGRFKKEYNGDQVIYGTQFDHMISTFPESAFRAGMRIAKYIDPAVYYD 120
S.c  FRGRSRKFQIQAEGRFKKEYNGNQVIFGTQFDHMISTFPESAFRAGMRIAKYIDPAVYYD 120
S.o  FRGRSRKFQIQAEGRFKKEYTGNQVIFGTQFDHMISTFPESAFRAGMRIAKYIDPAVYYD 120
     *:******************.*:***:********* ***********************

S.j  KNAKSPYIMSPFVACVNTLSAWPAPSRMEDAVISLVEADAQDSDSEPLPDVSDTFDETED 180
S.p  KYARSPYIMSPFVACVNTLSAWPAPSRLEDAVISLVEADSQESDTESLPEINDSSDVSLS 180
S.c  KFAKSPYIMSPFVACVNTLSAWPAPSRLDDAVISLVEADPQESDTDSLPDMSESLDDDLQ 180
S.o  KFAKSPYIMSPFVACVNTLSAWPAPSRLDDAVISLVEADPQESDTDSLPEMSESLDDDPQ 180
     * *:***********************::********** *:**:: **::.:: *   .

S.j  FTDFKKPEEKFEPKNNAEFMKTHLLTSPPAITVEGQD--ASEIEDSPRKPNDFDELSLIS 238
S.p  DLP----STNVTPKKTTTQIDVQTNIVTPPITVTAVPDSPNPPAATPATVADNDDLSIVS 236
S.c  PSI-------STPNKSPVGKSSSTRTPPPPIKITATQ--SPSPHTNVPSGTDHDNLSIVS 231
S.o  TPI-------SSPTKSSVSKSNLKTNTPPPITVTGSQ--LSPPHTNDPSRTDHDNLSLVS 231
                 *.:.   .        * *.: .           .  . * *:**::*

S.j  TDSGNTTT-PRHRRQRYWSFAGFNDNARFADLNKTSGPSHPQNL-------DVPSAVRPG 290
S.p  SDSGNTTA-PRKNRHRYWSFAGFSDSTRFSHLNVPAAIPDAPSVKTETSQNVSQLSVKSA 295
S.c  SDSGNTTADPNRNRHRYWSFAGFNDNTRFSHLNSASHTPETSTSRNKQRVETDPSLVDSI 291
S.o  SDSGNTTAY-QKGRHRYWSFAGFNNNTRFSHLNLAPHTPEVDESQKGQQNVPEQSLADSV 290
     :******:  .: *:********.:.:**:.**         .              .

S.j  SAGQYGDTGGYNSDEDEELLLHRHITNTHKDFSTHVKNVGLYHKQSLEDESEGTSS---- 346
S.p  A----TSLAPVEDDEDDELLLHRHITNKHKDFNLHVKQLGLLHKPSLDLEEGYIMDENYG 351
S.c  GSSLIRVSGEDADNDDDELILHRPITNRHKDFSRHVQQLGLLHKPSLDGD-GYV------ 344
S.o  GSSAIRRSGEDADSDDDELVLHRPITNRHKNFSVHVQQVGLLHKPSLDGD-GYV------ 343
     .      .    ..:*:**:*** *** **:*. **:::** ** **: :

S.j  --------ASDDTSADDERLQRYFTALELQVDKRSNEDDKKKKSDALHKLTHPSLKLGKK 398
S.p  KDVYKHGISNVPDDENEDDLQRYFTALEMQQDQKEQ-HKKSKNKDPFRKITHPSLHLGK- 409
S.c  NEFYDN---NSFDDENEEDLQRYFTALEMQSDKKH--YRKSKNKDPFHRITHPNLHIGK- 398
S.o  NEFYDD--DNSFDDGNEEDLQRYFTALEMQSDKKN--PRKPKNKDPFHRITHPNLHIGK- 398
              .   . ::: *********:* *::     * *:.* ::::***.*::**

S.j  LSAPKLIKRMSIRRKKKNHEDSTSDTSPTT-------ST--SNGSPVKHESKKSAPRRSL 449
S.p  FSTPKLIKRMSLRSKKSLRNDSKSDDVGNSTHRFSTASAASTSAVKTEKEKKMSAPRRSL 469
S.c  ISTPKIIKRMSLRSKKGLKSLNPESSSQTEAQRTSTSSM--SPPPVSKNEKKMSAPRRSL 456
S.o  ISTPKIIKRMSLRSKKGSKSSKQESSSQPETQRTSMSSM--SPPPVSKNEKKMSAPRRSL 456
     :*:**:*****:* **  :. . ..            *    :    ::*.* *******

S.j  DRLIRFGSIRRHHHHKHEDIKEPHR-----------------SDSVPIFPSRKSWSSSR 491
S.p  DKLIRIGSLHRHHH-------HHHKTDLIESDSGIEASESNRRKSDIFSFSGRNSFSVSR 522
S.c  DKLIRIGSLHRHHHHHHHHNQGPGSKPDQVESDSGFDLYKSES-KHHPELLLQRNSMSTDR 515
S.o  DKLIRIGSLHKHHHHHHHH--NQSKLDHPESDPGVDSYKNEG-RHQIESVPQRNSLSNDR 513
     *:***:**:::***          :                        .  *:* * .*

S.j  PYSTHSSGTSPRTSTESPLIESKYMSATQPPSRKSMSYVEGQSTYLSA-L---------- 540
S.p  PSSSHSTLSYANDSASSAVNVAG-ETGSLPPLREQTSITSGV--PPSNRLKKHVSTPEKI 579
S.c  PSSSQSTLTSPDDSLS-NPKVSG-ETGSLPPLREDTSPGESNGITSNPDHKEEPDKPGKN 573
S.o  PCSSPSTLTSPEGSISSTPKVSG-ETGSLPPLHEDTSPGENNGSTSKPAIKAEQDKPRNT 572
     * *: *: :    * .     :   :.: ** ::. *  ..     .

S.j  ---------PRAQEPSEEKIPEE-EGDTVSAKSAKLEQKHETPQSGNLLQVPNSRAVRRK 590
S.p  VEERSIDEVSQSNTPSSKQLPQSVDGKTVTAN-ANSTTVAKQPTSNVALTRPKPVRTATS 638
S.c  MGTPSLDISSHNP------SNDD-------SN-KKPGEVDNHLTVPTKLAKPSPSRTQSS 619
S.o  LQKPSLDMSSDNH------LQGS-------RN-GNTNDADNHLSVPKKPVKPVPSRTASH 618
                           .        :  :     :          *    .

S.j  KSGIPKPSKHIPSESTSLDACLGPWRFGNPKVDPIEDNSFIFGDLKSVKERRKYFSSSLA 650
S.p  QSKIPKPVKHIPSDSNNLDPQLGPWRFANPKVDPIEDNSFIFGEHKSVKERRKYFSSSKF 698
S.c  QSKIPKPCVHIPSESNSLDPQLGPFRFANPKVDPIEDNSFIFGEHKSVKDRRKYFSSSTD 679
S.o  QSKIPKPCVHIPSESNSLDPQLGPFRFANPKVDPIEDNSFIFGEHRSVKDRRKYFSSSEE 678
     :* ****  ****:*..**  ***:**.***************: :***:********

S.j  ARENFYFDRDVVYCMSFFAPYMDFNTFNLSFGPIRLNVNRSLNSEGKQPIRYMMREKENE 710
S.p  CRENFFYDKDVVYCMSFFSPHMDFNTFNLNIGPIRLNVYKHLNSDGHQPIRYMMRETDDE 758
S.c  TRADFYYDKDVVYCMSFFSPHMDFNRFNLNIGPIKLNVYRHLNSDGHQPIRYMMRETEDE 739
S.o  NRANFFYDKDVVYCMSFFSPHMDFNKFNLNIGPIKLNVFRHLNADGHQPIRYMMRETEDE 738
      * :*::*:*********:*:**** ***.:***:*** : **::*:*********.::*

S.j  KAIMFVVDFDLVEDDDKSVLEEQARNRELIAQRELGLGN 749
S.p  DAVMFVVEFDLLEDDDETVLAEQAKERERRKQT------ 791
S.c  HAIMFVVEFDLVEEDDEAVLLEQANERERRKH------- 771
S.o  NAIMFVIEFDLVEDDDEAVLAQQATERERRIN------- 770
     .*:***::***:*:**::** :** :**   :
```

Fig. S1. Duc1 is conserved among other *Schizosaccharomyces* species. Sequence alignment of Duc1 related proteins from other *Schizosaccharomyces* species. *S. pombe* (S.p), *Schizosaccharomyces japonicus* (S.j), *Schizosaccharomyces octpsporus* (S.o) and *Schizosaccharomyces cryophilus* (S.c). The DUF1769 domain segments are highlighted in gray and an FFAT-like sequence is highlighted in yellow. Residues conserved in all proteins are marked with an asterisk, similar residues are indicated with a colon and slightly similar residues are marked with a period.

Journal of Cell Science • Supplementary information

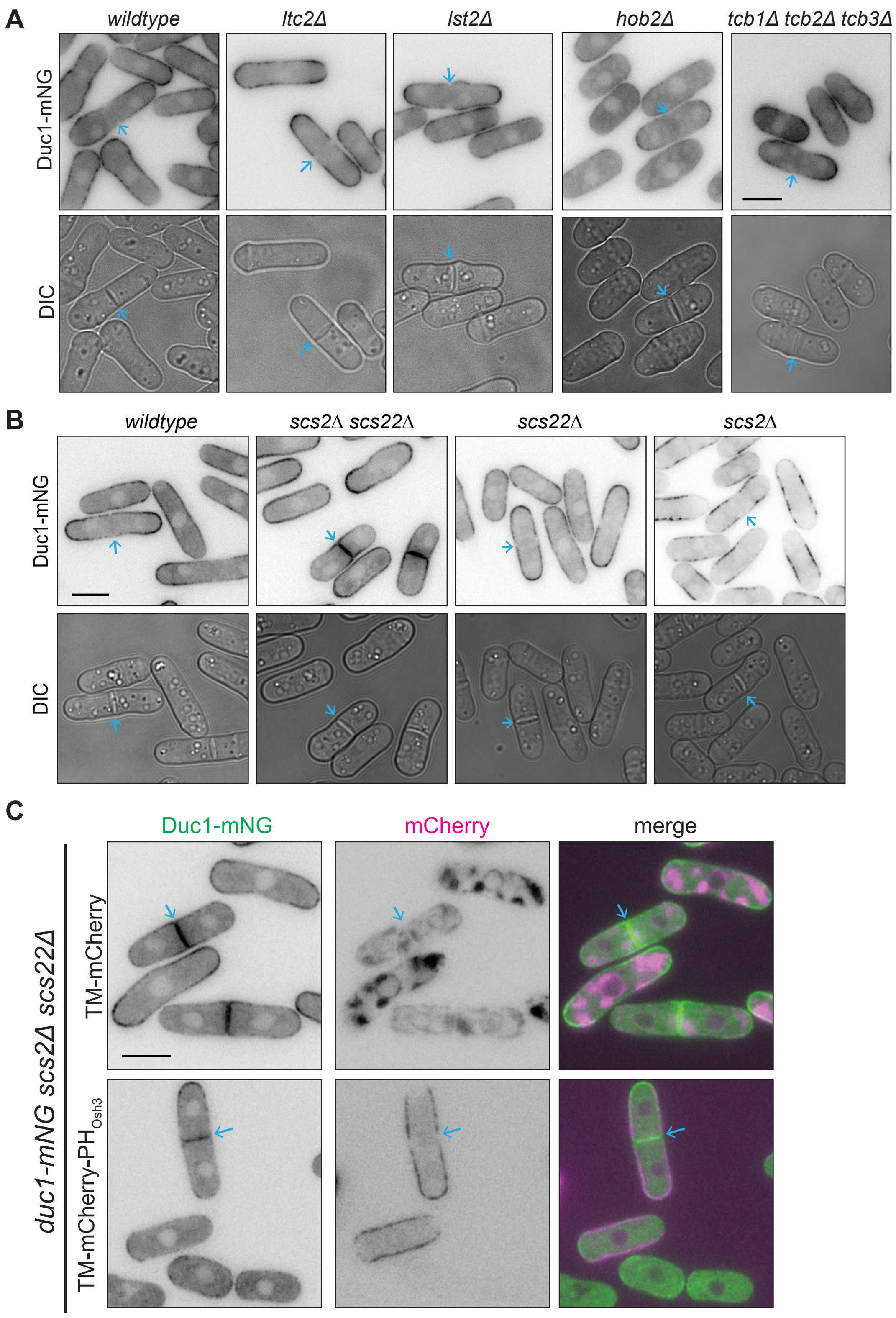

Fig. S2. Duc1 localization is not influenced by all ER-PM contact site proteins. (A-B) Live-cell imaging of cells expressing Duc1-mNG in the indicated genetic backgrounds. (C) Live-cell imaging of *duc1-mNG scs2*Δ *scs22*Δ cells expressing either TM-mCherry or TM-mCherry-PH_{Osh3} from the *nmt81* promoter. Expression was induced in media without thiamine for 24 hours prior to imaging. (A-C) Blue arrows indicated the position of a septum. Scale bars, 5 µm.

Journal of Cell Science • Supplementary information

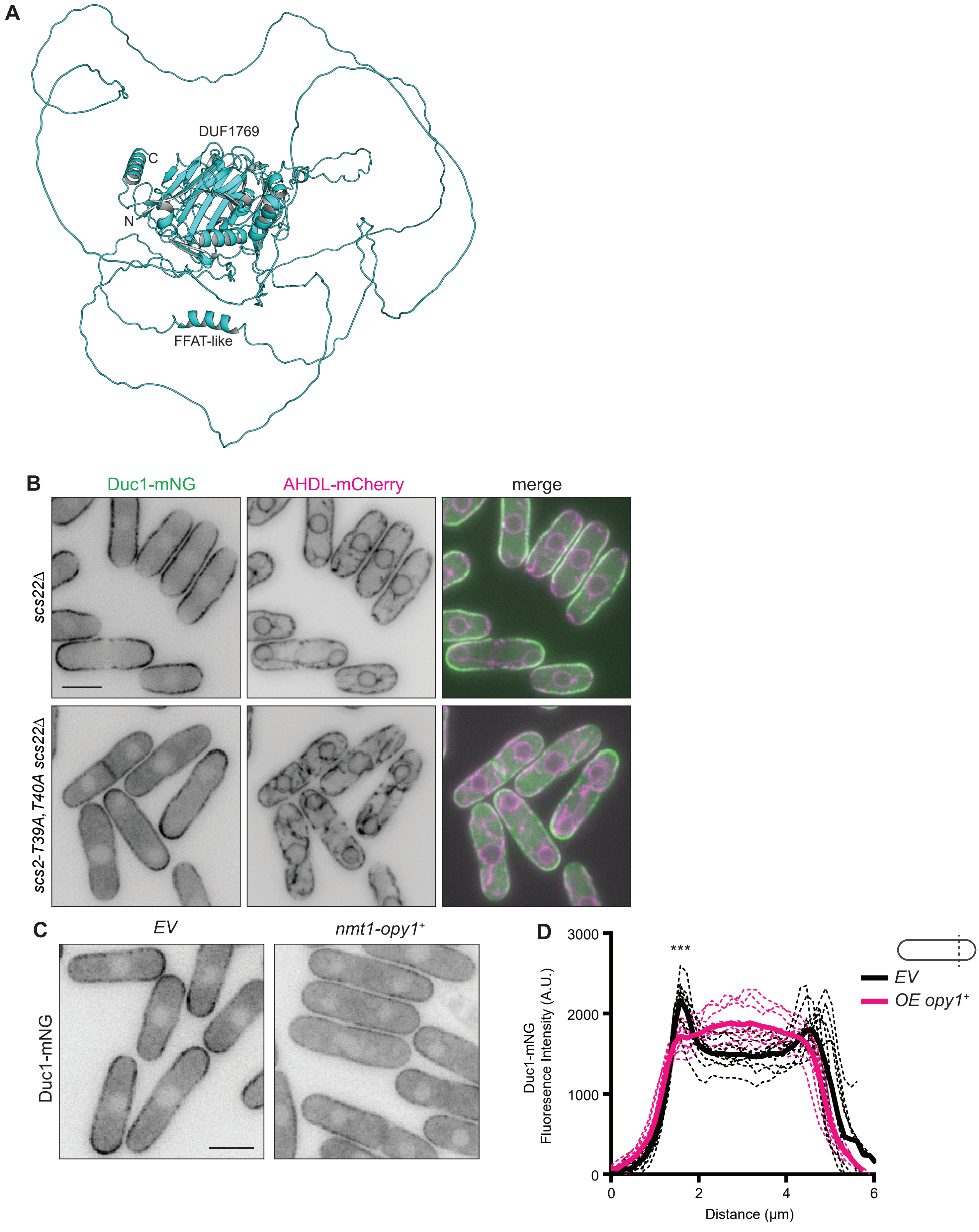

Journal of Cell Science • Supplementary information

Fig. S3. Duc1 exclusion of the cell division site it not dependent on ER-PM contacts. (A) AF predicted structure of the full Duc1 protein. The N- and C-termini, the DUF1769 domain and the FFAT-like sequence are indicated. (B) Live-cell imaging of *duc1-mNG AHDL-mCherry scs22∆* or *duc1-mNG AHDL-mCherry scs22∆ scs2-T39A,T40A* cells. (C) Live-cell imaging of cells expressing Duc1-mNG and expressing an empty vector (EV) or a vector with *nmt1-opy1⁺* induced for 24 hours prior to imaging. (D) Line scans of fluorescence intensity drawn across the short axis of 10 cells from each of the indicated strains. Solid lines represent the mean and dotted lines are the individual line traces. Data is from two biological replicates. For EV versus *nmt1-opy1⁺* expressing cells at first peak, 1.58 μm distance, $P<0.001$ (unpaired, two-tailed Student's t-test). ***$P<0.001$. Scale bars, 5 μm.

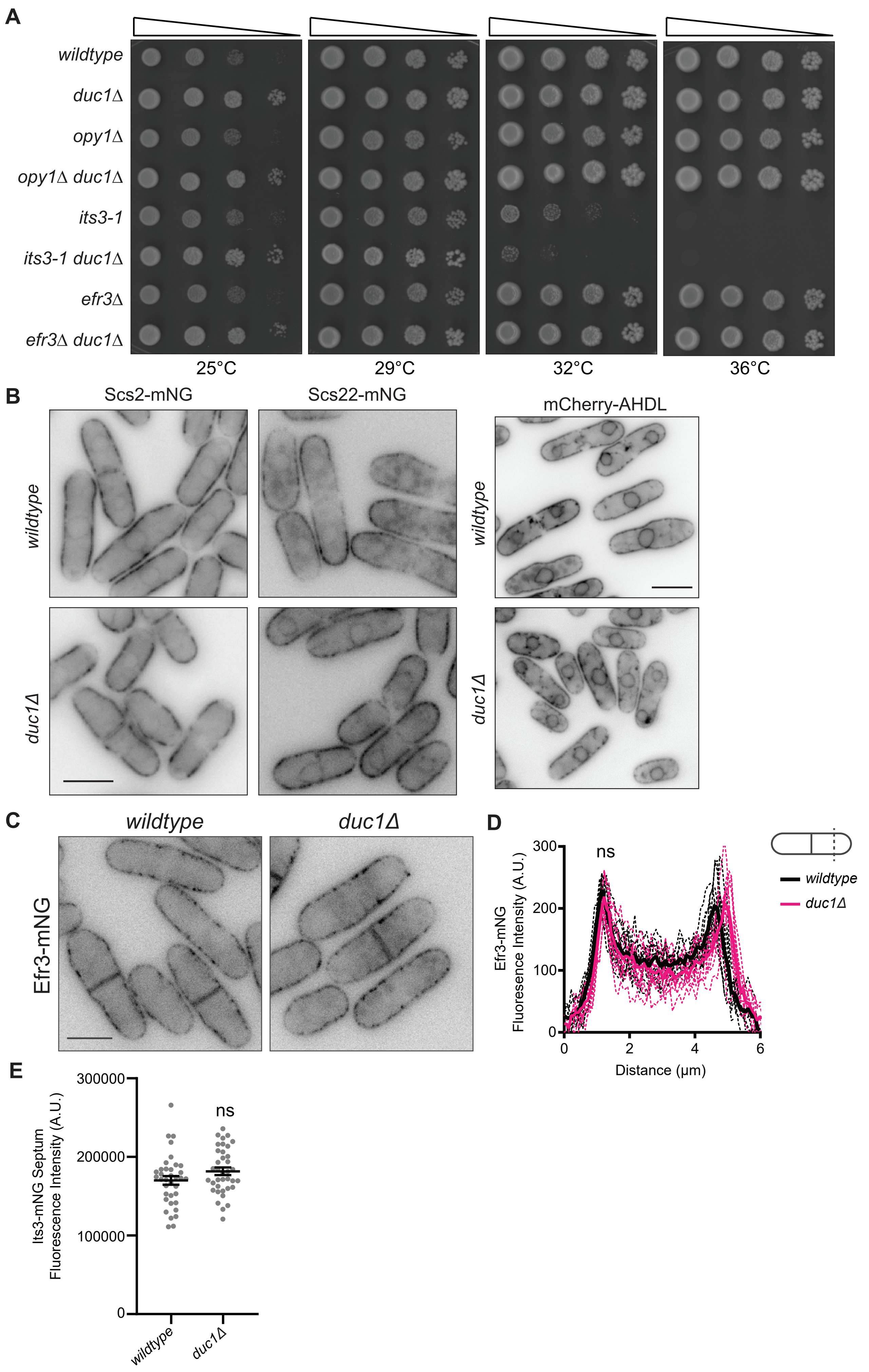

Journal of Cell Science • Supplementary information

Fig. S4. Genetic and microscopic analysis of *duc1Δ*. (A) The indicated strains were serially diluted ten-fold and spotted on YE plates at the indicated temperatures for 3 days prior to imaging. (B) Live-cell imaging of wildtype or *duc1Δ* cells expressing Scs2-mNG, Scs22-mNG or mCherry-AHDL. (C) Representative live-cell images of Efr3-mNG in wildtype or *duc1Δ* cells. (D) Line scans of fluorescence intensity drawn across the short axis of 10 cells from each of the indicated strains. Solid lines represent the mean and dotted lines are the individual line traces. Data is from two biological replicates. For Efr3-mNG signal in wild-type versus *duc1Δ* cells at first peak, 1.15 μm distance, is P=.377 (unpaired, two-tailed Student's t-test). (E) Quantification of the fluorescence intensity of its3-mNG at the septum in wildtype and *duc1Δ* cells. n⩾36 cells for each from three independent replicates. P=0.11 (unpaired, two-tailed Student's t-test). Scale bars, 5 μm.

Table S1. Proteins identified by LC-MS/MS in Its3-TurboID, Opy1-TurboID, and Myo2-TurboID. Shown in the table are the ORF numbers (ID), protein names, and protein descriptions. Also indicated are the Total Spectral Counts (TSC) of proteins from both the bait protein-TurboID and the control-TurboID, Only proteins with a minimum of two spectral counts are shown and proteins identified in control-TurboID are listed at the end of the table in grey-shaded text.

Available for download at
https://journals.biologists.com/jcs/article-lookup/doi/10.1242/jcs.262347#supplementary-data

Journal of Cell Science • Supplementary information

Table S2. *S. pombe* strains used in this study

**Figure 1**		
KGY19921	*its3-V5-TurboID:kanMX6 ade6-M210 ura4-D18 leu1-32* h^-	This study
KGY19922	*opy1-V5-TurboID:kanMX6 ade6-M210 ura4-D18 leu1-32* h^-	This study
KGY19896	*myo2-V5-TurboID:kanMX6 ade6-M210 ura4-D18 leu1-32* h^-	This study
KGY4485-2	*duc1-mNG:kanMX6 rlc1-mCherry:natMX6 sad1-mCherry:natMX6 ade6-M210 ura4-D18 leu1-32* h^+	This study
KGY4564-2	*its3-mCherry:natMX6 duc1-mNG:kanMX6 ade6-M210 ura4-D18 leu1-32* h^-	This study
KGY4563-2	*opy1-mCherry:natMX6 duc1-mNG:kanMX6 ade6-M210 ura4-D18 leu1-32* h^-	This study
**Figure 2**		
KGY6331-2	*duc1-mNG:kanMX6 leu1:pBip1-mCherry-AHDL ade6-M210 ura4-D18 leu1-32* h^+	This study
KGY6430-2	*duc1-mNG:kanMX6 leu1:pBip1-mCherry-AHDL scs2Δ::ura4*$^+$ *scs22Δ::ura4*$^+$ *ade6-M210 ura4-D18 leu1-32* h^+	This study
KGY7733-2	*duc1-mNG:kanMX6 rlc1-mCherry:natMX6 sad1-mCherry:natMX6 scs2Δ::ura4*$^+$ *scs22Δ::ura4*$^+$ *ade6-M210 ura4-D18 leu1-32* h^-	This study
**Figure 3**		
KGY8327-2	*scs22-mNG:hphMX6 duc1-mCherry:natMX6 ade6-M210 ura4-D18 leu1-32 h-*	This study
KGY7909-2	*tcb1-mNG:hphMX6 duc1-mCherry:natMX6 ade6-M210 ura4-D18 leu1-32 h-*	This study
KGY7810-2	*hob2-mNG:hphMX6 duc1-mCherry:natMX6 ade6-M210 ura4-D18 leu1-32 h-*	This study
KGY7794-2	*ltc2-mNG:hphMX6 duc1-mCherry:natMX6 ade6-M210 ura4-D18 leu1-32 h-*	This study
KGY7809-2	*ist2-mNG:hphMX6 duc1-mCherry:natMX6 ade6-M210 ura4-D18 leu1-32 h-*	This study
KGY7579-2	*leu1:pBip1-mCherry-AHDL rlc1-mNG:hphMX6 sid4-mNG:kanMX6 ade6-M210 ura4-D18 leu1-32* h^+	This study
**Figure 4**		
KGY291-2	*duc1-mNG:kanMX6 ade6-M210 ura4-D18 leu1-32* h^-	This study
KGY9128-2	*duc1-mNG:kanMX6 scs22Δ::ura4*$^+$ *scs2-T39A,T40A ade6-M210 ura4-D18 leu1-32* h^-	This study
KGY9028-2	*duc1-Y374A,F375A-mNG:kanMX6 ade6-M210 ura4-D18 leu1-32* h^-	This study
**Figure 5**		
KGY7582-2	*duc1-mNG:kanMX6 efr3Δ::kanMX6 ade6-M210 ura4-D18 leu1-32* h^+	This study
KGY581-2	*duc1-mNG:kanMX6 its3-1 ade6-M210 ura4-D18 leu1-32* h^-	This study
KGY1879-2	*opy1-mNG:kanMX6 ade6-M210 ura4-D18 leu1-32* h^+	Lab stock

Journal of Cell Science • Supplementary information

KGY246	*ade6-M210 ura4-D18 leu1-32 h*$^{-}$	Lab stock
**Figure 6**		
KGY338-3	*duc1Δ::ura4*$^{+}$ *ade6-M210 ura4-D18 leu1-32 h*$^{-}$	This study
KGY269-2	*GFP-2xPH(Plc):leu1*$^{+}$ *ade6-M210 ura4-D18 leu1-32 h*$^{-}$	Lab stock
KGY19217	*GFP-P4C(SidC):leu1*$^{+}$ *ade6-M210 ura4-D18 leu1-32 h*$^{+}$	Lab stock
KGY1401-3	*GFP-2xPH(Plc):leu1*$^{+}$ *duc1Δ::ura4*$^{+}$ *ade6-x ura4-D18 leu1-32 h*$^{-}$	This study
KGY4506-2	*GFP-P4C(SidC):leu1*$^{+}$ *duc1Δ::ura4*$^{+}$ *ade6-M21x ura4-D18 leu1-32 h*$^{+}$	This study
KGY19002	*rlc1-mNG:hphMX6 sid4-mNG:kanMX6 ade6-M210 ura4-D18 leu1-32 h*$^{-}$	Lab stock
KGY7732-2	*duc1Δ::ura4*$^{+}$ *rlc1-mNG:hphMX6 sid4-mNG:kanMX6 ade6-M210 ura4-D18 leu1-32 h*$^{+}$	This study
KGY243-2	*its3-mNG:hphMX6 ade6-M210 ura4-D18 leu1-32 h*$^{-}$	Lab stock
KGY584-2	*its3-mNG:hphMX6 duc1Δ::ura4*$^{+}$ *ade6-M210 ura4-D18 leu1-32 h*$^{-}$	This study
**Figure 7**		
KGY488-2	*its3-mNG:hphMX6 scs2Δ::ura4*$^{+}$ *scs22Δ::ura4*$^{+}$ *ade6-M210 ura4-D18 leu1-32 h*$^{-}$	This study
KGY2839-2	*GFP-2xPH(Plc):leu1*$^{+}$ *scs2Δ::ura4*$^{+}$ *scs22Δ::ura4*$^{+}$ *ade6-x ura4-D18 leu1-32 h*$^{-}$	This study
KGY2825-2	*scs2Δ::ura4*$^{+}$ *scs22Δ::ura4*$^{+}$ *rlc1-mNG:hphMX6 sid4-mNG:kanMX6 ade6-M210 ura4-D18 leu1-32 h*$^{+}$	This study
**Figure S2**		
KGY8139-2	*duc1-mNG:kanMX6 ltc2Δ::kanMX6 ade6-M210 ura4-D18 leu1-32 h*$^{+}$	This study
KGY9177-2	*duc1-mNG:kanMX6 ist2Δ::kanMX6 ade6-M210 ura4-D18 leu1-32 h*$^{+}$	This study
KGY8576-2	*duc1-mNG:kanMX6 hob2Δ::ura4*$^{+}$ *ade6-M210 ura4-D18 leu1-32 h*$^{+}$	This study
KGY8222-2	*duc1-mNG:kanMX6 tcb1Δ::kanMX6 tcb2Δ::ura4*$^{+}$ *tcb3Δ::kanMX6 ade6-M210 ura4-D18 leu1-32 h*$^{-}$	This study
KGY7865-2	*duc1-mNG:kanMX6 scs2Δ::ura4+ ade6-M210 ura4-D18 leu1-32 h*$^{+}$	This study
KGY7866-2	*duc1-mNG:kanMX6 scs22Δ::ura4*$^{+}$ *ade6-M210 ura4-D18 leu1-32 h*$^{+}$	This study
KGY6432-2	*duc1-mNG:kanMX6 scs2Δ::ura4*$^{+}$ *scs22Δ::ura4*$^{+}$ *ade6-M210 ura4-D18 leu1-32 h*$^{-}$	This study
**Figure S3**		
KGY9217-2	*duc1-mNG:kanMX6 leu1:pBip1-mCherry-AHDL scs22Δ::ura4*$^{+}$ *ade6-M210 ura4-D18 leu1-32 h*$^{+}$	This study
KGY9227-2	*duc1-mNG:kanMX6 leu1:pBip1-mCherry-AHDL scs22Δ::ura4*$^{+}$ *scs2-T39A,T40A ade6-M210 ura4-D18 leu1-32 h*$^{+}$	This study
**Figure S4**		

KGY6369-2	*its3-1 ade6-M21X ura4-D18 leu1-32* h^-	Lab stock
KGY900-3	*duc1Δ::ura4*$^+$ *its3-1 ade6-M210 ura4-D18 leu1-32* h^-	This study
KGY15603	*efr3Δ::kanMX6 ade6-M210 ura4-D18 leu1-32* h^+	Lab stock
KGY7582-2	*duc1Δ::ura4*$^+$ *efr3Δ::kanMX6 ade6-M210 ura4-D18 leu1-32* h^-	This study
KGY250-2	*opy1Δ::kanMX6 ade6-M210 ura4-D18 leu1-32* h^-	Lab stock
KGY4510-2	*opy1Δ::kanMX6 duc1Δ::ura4*$^+$ *ade6-M210 ura4-D18 leu1-32* h^+	This study
KGY7430-2	*scs2-mNG:kanMX6 ade6-M210 ura4-D18 leu1-32* h^-	This study
KGY8326-2	*scs2-mNG:kanMX6 duc1Δ::ura4*$^+$ *ade6-M210 ura4-D18 leu1-32* h^-	This study
KGY7695-2	*scs22-mNG:kanMX6 ade6-M210 ura4-D18 leu1-32* h^+	This study
KGY8324-2	*scs22-mNG:kanMX6 duc1Δ::ura4*$^+$ *ade6-M210 ura4-D18 leu1-32* h^-	This study
KGY8325-2	*leu1:pBip1-mCherry-AHDL duc1Δ::ura4*$^+$ *ade6-M210 ura4-D18 leu1-32* h^-	This study
KGY17908	*efr3-mNG:kanMX6 ade6-M210 ura4-D18 leu1-32* h^-	Lab stock
KGY9032-2	*efr3-mNG:kanMX6 duc1Δ::ura4*$^+$ *ade6-M210 ura4-D18 leu1-32* h^-	This study

Journal of Cell Science • Supplementary information
